# Endoplasmic Reticulum Oxidative Stress Triggers Tgf-Beta-Dependent Muscle Dysfunction by Accelerating Ascorbic Acid Turnover

**DOI:** 10.1038/srep40993

**Published:** 2017-01-20

**Authors:** Diego Pozzer, Mariagrazia Favellato, Marco Bolis, Roberto William Invernizzi, Francesca Solagna, Bert Blaauw, Ester Zito

**Affiliations:** 1 IRCCS-Istituto di Ricerche Farmacologiche Mario Negri, Milan, Italy; 2 Venetian Institute of Molecular Medicine, 35100, Padua, Italy; 3 Department of Biomedical Science, University of Padova, 35100, Padua, Italy; 4 Dulbecco Telethon Institute at IRCCS-Istituto di Ricerche Farmacologiche Mario Negri, Milan, Italy

## Abstract

Endoplasmic reticulum (ER) and oxidative stress are two related phenomena that have important metabolic consequences. As many skeletal muscle diseases are triggered by oxidative stress, we explored the chain of events linking a hyperoxidized ER (which causes ER and oxidative stress) with skeletal muscle dysfunction. An unbiased exon expression array showed that the combined genetic modulation of the two master ER redox proteins, selenoprotein N (SEPN1) and endoplasmic oxidoreductin 1 (ERO1), led to an SEPN1-related myopathic phenotype due to excessive signalling of transforming growth factor (TGF)-beta. The increased TGF-beta activity in the genetic mutants was caused by accelerated turnover of the ER localized (anti-oxidant) ascorbic acid that affected collagen deposition in the extracellular matrix. In a mouse mutant of SEPN1, which is dependent on exogenous ascorbic acid, a limited intake of ascorbic acid revealed a myopathic phenotype as a consequence of an altered TGF-beta signalling. Indeed, systemic antagonism of TGF-beta re-established skeletal muscle function in SEPN1 mutant mice. In conclusion, this study sheds new light on the molecular mechanism of SEPN1-related myopathies and indicates that the TGF-beta/ERO1/ascorbic acid axis offers potential for their treatment.

The endoplasmic reticulum (ER) is the compartment in which the proteins destined for the secretory pathway and cell surface are folded. It is predicted that one-third of the proteins encoded by the human genome enter the secretory pathway, and their misfolding generates ER stress. Nutrient deprivation, changes in ER calcium content, and oxidative stress interfere with normal protein folding and, by triggering ER stress, lead to the activation of the unfolded protein response (UPR)^1^.

Although basal UPR signaling occurs in normal skeletal muscle an excess of UPR can be deleterious^2^. Indeed, many muscle pathologies are associated with ER stress and UPR^3^,^4^. Furthermore, a considerable increase in oxidative stress and activation of the UPR is associated with various acquired muscle wasting conditions, such as aging, cancer and genetic myopathies^5^,^6^, but the mechanism by means of which hyperoxidation interferes with skeletal muscle function is still unclear.

Genetic analyses of ER stress receptor inactivation showed the importance of the UPR as a survival pathway for ER-stressed cells^7^. However, closer scrutiny has revealed evidence favouring a more nuanced view of the relationship between the UPR and cell survival and suggested that ER hyperoxidation can lead to cell dysfunction and death through a maladaptive UPR.

The transcription factor C/EBP homologous protein (CHOP) is highly activated during ER stress and, despite its well-established status as a UPR marker, its deletion protects stressed cells against death, which suggests that it has evolved to associate ER stress with cell death^8^.

ERO1, the main protein disulfide oxidase of the ER that generates H_2_O_2_, is a transcriptional target of CHOP. A failure to activate ERO1 expression can explain the less oxidising conditions observed in the ER of stressed CHOP knock-out cells, and may contribute to their ability to survive unusually high levels of ER stress^9^,^10^. Accordingly, the results of *Caenorhabditis elegans* experiments suggest that inhibiting ERO1 under conditions of ER stress by lowering the content of reactive oxygen species (ROS) increases the fitness and the life span of this model, thus suggesting that ERO1 may contribute to cell death during ER stress^11^,^12^. However, the pathophysiological consequences of ERO1-induced hyperoxidation are still unknown in vertebrates.

Ultrastructural studies of skeletal muscle suggest its sarcoplasmic reticulum may be prone to hyperoxidation as it is involved in stable structural and functional interactions with mitochondria in what is known as the mitochondria-associated membrane (MAM)^13^.

Skeletal muscle fibers are particularly abundant in mitochondria, which are the major source of ROS because of the activity of the mitochondrial respiratory chain^14^. The ROS generated in mitochondria can therefore move into the ER through the MAM, hyperoxidizes the ER, and elicit an UPR. In this context, the UPR-mediated up-regulation of ERO1 may induce the production of additional H_2_O_2_, and lead to maladaptation and dysfunction.

Mice null for hexose-6-phosphate dehydrogenase, an enzyme that produces NADPH and therefore may counteract the ER hyperoxidation, show upregulation of ER stress markers in skeletal muscle and severe myopathy, supporting the hypothesis that ER hyperoxidation leads to ER stress and muscle dysfunction^15^.

Further evidence suggesting that ER hyperoxidation plays a special role in skeletal muscle is the myopathic phenotype of SEPN1 loss of function. SEPN1, a member of the selenocysteine-containing protein family that has a reductase function is localised in the ER lumen and ubiquitously expressed throughout the body. However, despite its ubiquitous expression, the loss-of-function mutation leads to rare early-onset recessive neuromuscular disorders. It has also been shown that SEPN1 knock-out (KO) mice are particularly sensitive to ER hyperoxidation as we have previously found that the over-expression of ERO1 is sufficient to compromise muscle function in SEPN1 KO but not in wild-type (WT) mice^16^.

The prominence of a muscle phenotype associated with loss-of-function SEPN1 not only shows that ER hyperoxidation plays a special role in skeletal muscle, but also suggests that working on a redox-uncompensated SEPN1 KO background may help to reveal the molecular determinants that link excessive ERO1 activity and consequent ER hyperoxidation with skeletal muscle function. We therefore used an unbiased exon expression array to identify the pathways that are deregulated by both SEPN1 KO and ERO1 over-expression in skeletal muscle. The pathway analysis indicated that TGF-beta stands out among the deregulated pathways, and functional analysis revealed a causal link between TGF-beta hyperactivation and the myopathic phenotype. A suitable cell system and a mouse model devoid of SEPN1 and dependent on exogenous ascorbic acid both showed that, by lowering collagen deposition in the extra-cellular matrix, an increased intra-cellular turnover of ascorbic acid hyperactivated TGF-beta.

## Results

### ERO1 over-expression in SEPN1 KO muscle affects muscle function by hyper-activating the TGF-beta pathway

Unlike humans, in whom loss of function SEPN1 gives rise to SEPN1-related myopathies, SEPN1 KO mice are somehow protected, and show no gross alterations in muscle histology or strength^17^,^18^. We therefore decided to add a further redox stress to the SEPN1 deficit in murine muscle by inducing the over-expression of ERO1.

**Figure 1:**
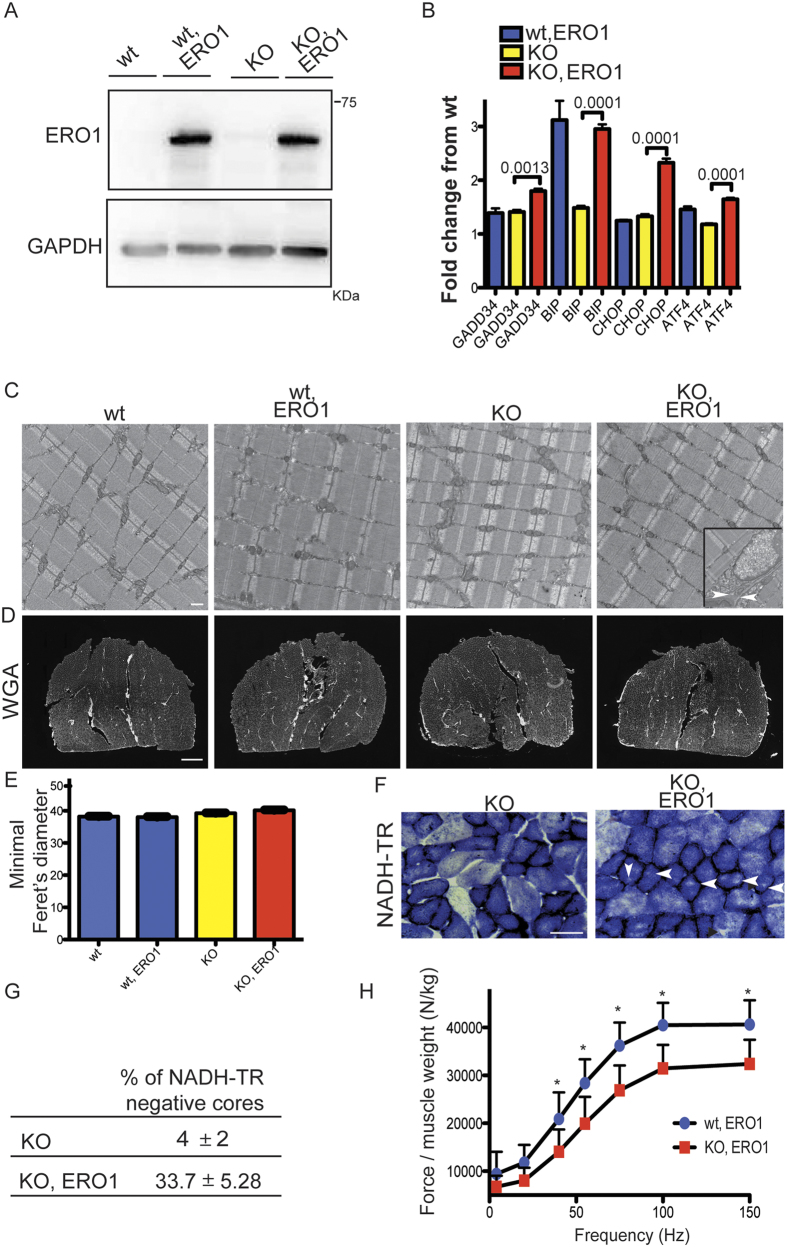
Intramuscular AAV2.1-ERO1 injection reduces specific muscle strength in SEPN1 KO mice. (**A**) ERO1α and GAPDH representative immunoblot of proteins extracted from the gastrocnemii of wild-type and SEPN1 KO mice transduced with *AAV2.1-ERO1α* (wt, ERO1 and KO, ERO1) or mock *AAV2.1-GFP* (wt and KO). (**B**) Abundance of UPR markers mRNAs (fold-change in relation to WT) measured by quantitative real time PCR in cDNA from gastrocnemii (n = 4 per each group). The bar graphs show mean values ± SEM; the differences were examined using a 2-tailed unpaired Student’s *t* test. P values are indicated on the graph. (**C**) Representative electron micrographs (scale bar: 1 μm) of gastrocnemii. The inset indicates a representative cell with dilation of the ER (the two white arrowheads delimit the ER dilation). (**D**) Representative histology of wheat-germ agglutinin (WGA) stain of gastrocnemii. (**E**) Minimal Feret’s diameter (μm) of gastrocnemii (n = 4 muscles and 6000 fibers counted per condition) (scale bar: 50 μm). (**F**) Representative histology of NADH-TR indicates minicores in KO, ERO1 (white arrowheads) (scale bar: 500 μm). (**G**) Table indicating the percentage of minicore-like structures in type 1 fibers of gastrocnemii as revealed by NADH-TR labelling (n = 6, average value from 100 type 1 fibers). (**H**) The frequency curve of the *in vivo* force measurements normalized to the gastrocnemius weight showing that KO, ERO1 mice are weaker than wt, ERO1 mice (n = 10 wt, ERO1 and n = 15 KO, ERO1). Mean values ± SEM; the differences were examined using two-way ANOVA followed by Bonferroni’s post-test correction. P = 0.01 at 40 Hz; P = 0.001 at 55, 75, 100 and 150 Hz.

In order to test the effects of ERO1 over-expression on the muscle phenotype of SEPN1 KO mice, one-month-old SEPN1 KO mice were intramuscularly injected with AAV2/1-*ERO1*α (an adeno-associated virus driving ERO1α), hereafter reffered to as ERO1, in three sites of the right gastrocnemius muscle, and AAV2/1-*GFP* or vehicle alone was injected into the contralateral muscle. The animals were sacrificed four-six weeks after injection, and the levels of ERO1 expression were analysed by means of Western blotting (Fig. 1A).

To examine whether ERO1 overexpression, by hyperoxidizing the ER, as previously shown by the higher ratio between oxidised and total glutathione, induced ER stress and UPR in skeletal muscle, we investigated the expression of some indicators of ER stress in gastrocnemius muscles of *GFP-*injected and *ERO1-*injected SEPN1 KO mice and saw further induction of these indicators in *ERO1-*injected SEPN1 KO muscle compared to *GFP-*injected SEPN1 KO (Fig. 1B).

However, the ER stress triggered by ERO1 overexpression in SEPN1 KO muscle was not accompanied by any obvious signs of alteration of the muscle ultrastructure, except some rare cells with ER dilation (Fig. 1C), and overt muscle atrophy (Fig. 1D and E), thus confirming that the over-expression of ERO1 did not alter the overall muscle structure.

Importantly, quantitative analysis of the gastrocnemii of ERO1*-*injected SEPN1 KO mice revealed minicore-like structures, an hallmark of SEPN1-related myopathies, indicating regions of mitochondria depletion, in more than 30% of the type 1 muscle fibers (Fig. 1F and G).

In order to understand if the presence of minicores was an indication of compromised muscle function, we evaluated the *in vivo* force production of the gastrocnemius muscle in WT, SEPN1 KO or ERO1*-*injected WT and SEPN1 KO mice. Interestingly, we observed a selectively reduced muscle tension at all stimulation frequencies in the *ERO1-injected* SEPN1 KO gastrocnemius, while ERO1 overexpression in WT muscles did not lead to a reduction in normalized muscle force (Fig. 1H). These findings show that the over-expression of ERO1 in SEPN1 KO muscles impairs muscle strength and creates a muscle dysfunction similar to that observed in SEPN1-related myopathies.

**Figure 2:**
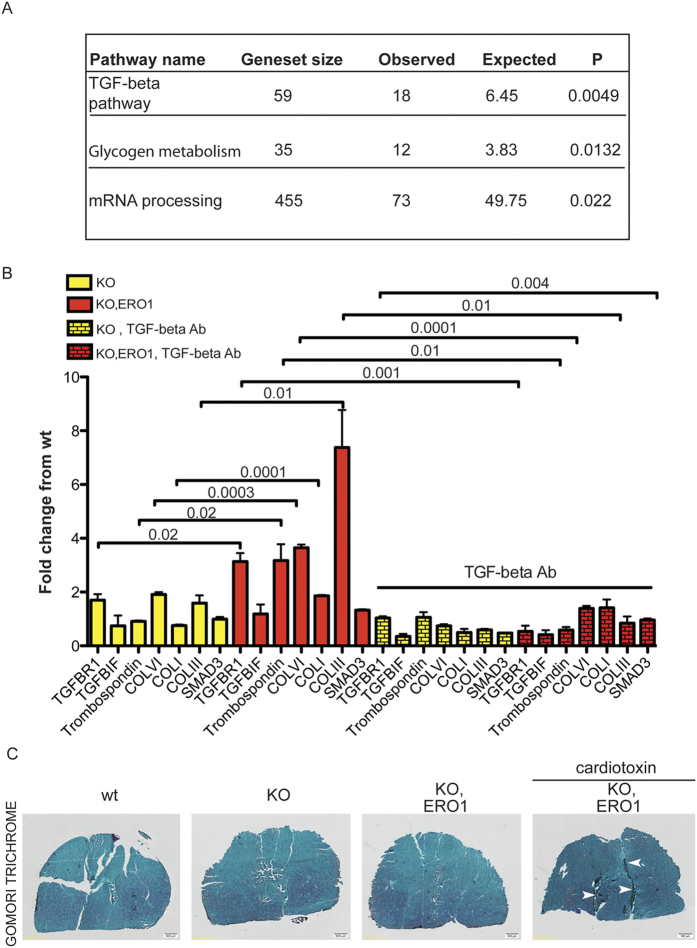
TGF-beta pathway is hyperactive in SEPN1 KO after the intramuscular injection of AAV2.1-ERO1 . (**A**) Pathway analysis of the genes regulated by the KO and KO, ERO1 axis. Affymetrix Mouse GeneChip Gene ST 2.1 arrays were used to identify the gastrocnemius mRNAs that were increased by KO (n = 2) and KO, ERO1 (n = 2) in comparison with WT (n = 2) and WT, ERO1 (n = 2). Statistical significance was determined using hypergeometric testing and adjusted by false discovery rate (P adjusted < 0.05). (**B**) Abundance of TGF-beta pathway mRNAs (fold-change in relation to WT) measured using quantitative real-time PCR in cDNA from gastrocnemii, and after the intraperitoneal injection of a TGF-β neutralising antibody (n = 3 per group). The bar graphs show mean values ± SEM; the differences between KO and KO, ERO1 and between KO, ERO1 and KO, ERO1 after the injection of the TGF-β neutralising antibody were examined using a 2-tailed unpaired Student’s *t* test. P values are indicated on the graph and in this condition of multiple tests (14 comparisons) and P value threshold of 0.05 the false discovery rate is calculated to 7%. (**C**) Light micrographs of sections of gastrocnemius stained with modified Gomori’s Trichrome. The arrows in the muscle injected with cardiotoxin indicate the area of fibrosis that are not noticed are not seen in the non-injected muscles (n = 4 per each group).

In an attempt to define the transcriptional programme regulated by SEPN1 and ERO1, we used unbiased trascriptomic profiling of the gastrocnemii of WT and SEPN1 KO mice with or without the injection of ERO1. Two-way analysis of variance (ANOVA) was used to compute statistical significance between experimental conditions (genotype/ERO1 injection) and their interaction. We selected the genes that were differentially expressed in SEPN1 KO and WT animals (p_KO_ < 0.01), and then used pathway enrichment analysis to reveal the strong enrichment of the TGF-beta, a cytokine resident in the ECM whose hyperactivation is a known cause of myopathies and other muscle disease^19^ (Fig. 2A and Sup. Fig. 1 A and B). Moreover, the quantitative real-time polymerase chain reaction (qRT-PCR) analysis of the gastrocnemii indicated further up-regulation of the TGF-beta target genes in the ERO1-injected SEPN1 KO muscle in comparison with the mock-injected SEPN1 KO gastrocnemii (Fig. 2B).

However, ERO1*-*injected SEPN1 KO did not show an overt skeletal muscle fibrosis (i.e. augmented collagen content in the ECM), that usually accompanied other pathological muscle phenotype with an increased TGF-beta signalling^19^ (Fig. 2C).

### Faster ascorbic acid turnover impairs collagen content in ECM and hyperactivates TGF-beta signalling in SEPN1 defective myoblasts

**Figure 3:**
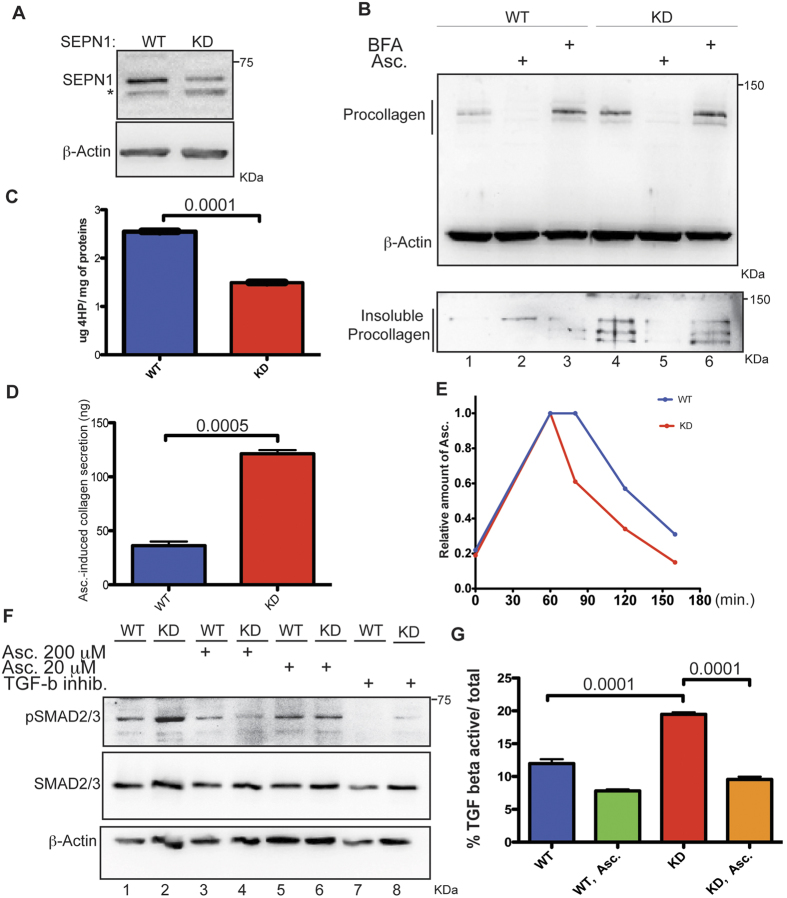
A reduced SEPN1 level, by accelerating ascorbic acid turnover, hyperactivates the TGF-beta pathway. (**A**) Immunoblot of SEPN1 and β-Actin in C2C12 after transduction with a lentivirus carrying an irrelevant insert (WT) or a short hairpin RNA directed to mouse SEPN1 (KD). KD cells were already tested and used in^16^.* background band. (**B**) Immunoblot of type I procollagen and β-Actin. Where noted cells were exposed to BFA (2 μg/mL) for 6 hours to inhibit export of procollagen from the ER and Ascorbic acid (Asc.) at 200 μM. The lower panel reports the relative content of SDS-insoluble procollagen. (**C**) 4-hydroxyproline content of lysates of BFA treated cells normalized to the total content of intracellular proteins (n = 3 per each group). The bar graphs indicate means ± SEM, differences were examined using a 2-tailed unpaired Student’s *t* tests and P value is indicated on the graph. (**D**) Bar diagram of collagen secreted into the conditioned media of cells treated for three days with 200 μM Asc. and normalized to μg of intracellular proteins (n = 3 per each group). The bar graphs indicate means ± SEM, differences were examined using a 2-tailed unpaired Student’s *t* tests and P value is indicated on the graph. (**E**) Asc. metabolism in WT and KD cells. The cells were cultured in presence of dehydroascorbic acid (DHA) and the intracellular metabolism of the ascorbic acid examined by HPLC. Briefly, cells are supplemented with 25 μM of DHA for half hour and then the ascorbic acid cellular content is measured at different time points after withdrawal of the oxidized vitamin. The height of the peaks at any time point is plotted after the arbitrary value of 1 had been assigned to the highest peak. (**F**) Immunoblots of SMAD2/3 phosphorylation, total SMAD2/3 and β-Actin from cells treated with ascorbic acid and SB431542, a specific TGFβ inhibitor. (**G**) Bar graphs representing the percentage of the ratio between active and total TGFβof cells treated with 20 μM Asc. (n = 3 per each group). The bar graphs indicate means ± SEM, differences were examined using a one-way ANOVA test and Bonferroni’s post-test. P values are indicated on the graph.

In order to characterise the chain of events that can connect a hyperoxidised ER to a hyperactive TGF-beta signalling that does not cause fibrosis, we used a cell system of SEPN1 knock-down (SEPN1 KD) C2C12 myoblasts^16^ (Fig. 3A).

TGF-beta belongs to a family of cytokines that transduce their signals through the SMAD cascade. Ligand-activated TGF-beta receptors induce the phosphorylation of SMAD2 and SMAD3, which activate target gene responses in the nucleus^20^. The SEPN1 KD myoblasts had higher levels of phosporylated SMAD2/3 (pSMAD2/3), thus confirming that this cell system resembles the mutant muscle model in terms of TGF-beta hyperactivity (Fig. 3F lanes 1 and 2).

As altered TGF-beta signalling is often triggered by defects in ECM components, and we have previously observed defective collagen biogenesis in another mouse model of hyperoxidised ER, we investigated whether collagen, a major ECM protein and its intracellular precursor procollagen folded inside the ER may be responsible for the defect in SEPN1 KD cells^21^,^22^.

Remarkably, SEPN1 KD cells had high steady-state levels of procollagen I, and this accumulation was worsened by the transient over-expression of ERO1 (Fig. 3B and Sup Fig. 2A). Moreover, a fraction of the procollagen was recovered in the SDS-insoluble complexes and thus was probably unfolded (Fig. 3B lane 4 in the lower panel). These observations indicate defective procollagen folding in SEPN1 KD cells.

Type I collagen is synthesised as precursor procollagen inside the ER where is hydroxylated by the ER enzymes proline and lysine hydroxylases, and then after being processed to mature collagen in the post-ER compartments, it is ready to be secreted and deposited in the ECM^23^.

Accordingly, we found that procollagen I, which was trapped in the ER after treatment with brefaldin A (BFA, a drug that interferes with the exit of proteins from the ER) was less hydroxylated in the SEPN1 KD cells (Fig. 3B lane 6 and 3 C). As ascorbic acid (a reducing agent in cells but also a co-factor that maintains the activity of ER-localised proline 4-hydroxylases) can affect the activity of proline 4 hydroxylation by blocking procollagen secretion and causing its retention in the ER^24^, we investigated the accumulation of procollagen in SEPN1 KD cells treated with ascorbic acid. The ascorbic acid reversed the retention of both soluble and SDS-insoluble procollagen, and simultaneously increased the levels of soluble collagen being secreted (Fig. 3B lane 5 and 3D). The responsiveness of the mutant cells to ascorbic acid supplementation suggested a functional deficiency in cells’ ascorbic acid content.

We have previously observed that abundant (millimolar concentrations in most tissues^25^) and well-placed ER-resident ascorbic acid may also be involved in a second electron redox reaction that, by relieving ER proteins of their hyperoxidised state, converts ascorbate into an unstable oxidised dehydroascorbate derivative^22^,^26^. The dehydroascorbate undergoes a rapid hydrolytic process that produces the 2, 3-diketo-l-Gulonate (2, 3-DKG) derivative, which cannot be reconverted and so leads to a net loss of ascorbic acid^27^.

In order to investigate whether ascorbic acid is involved in this second redox reaction in cells with defective SEPN1, we explored the metabolism of ascorbic acid in SEPN1 KD cells using quantitative high-performance liquid chromatography (HPLC)-based method that selectively measures reduced ascorbic acid^28^ and (Sup Fig. 2B). After a pulse of ascorbic acid (vitamin C), the decay of the vitamin was faster in the SEPN1 KD cells than in the WT cells (Fig. 3E) suggesting role of ascorbic acid additional to that of co-factor of proline-4-hydroxylase in SEPN1 KD cells.

We subsequently investigated whether ascorbic acid supplementation was sufficient to normalise TGF-beta activity in SEPN1 KD myoblasts, and found that it reduced the phosporylation of SMAD2/3 to a value that was similar to that observed in the WT cells (as shown by Western blotting), and also reduced the general activity of TGF-β as shown by the TGF-beta responsive luciferase reporter assay (Fig. 3F lanes 4 and 6 and 3 G). These findings therefore indicate a direct relationship between cell ascorbic acid content, ECM collagen levels, and TGF-beta activity.

In brief, possibly by skewing the ER function of ascorbic acid from that of a co-factor of collagen hydroxylases to that of a general reductant, a hyperoxidized ER depletes ER-resident hydroxylases of ascorbic acid and consequently reduces the collagen content of the ECM hyperactivating the TGF-beta pathway.

### TGF-beta inhibition rescues muscle weakness in SEPN1 KO mice

In order to investigate the cause-effect relationship between TGF-beta hyperactivity and the development of myopathy in *ERO1-*injected SEPN1 KO mice, we systemically antagonised TGF-beta *in vivo* by intraperitoneally injecting 1 mg/kg of neutralising antibody from the age of five weeks, as has been done when treating other myopathic states^19^.

**Figure 4:**
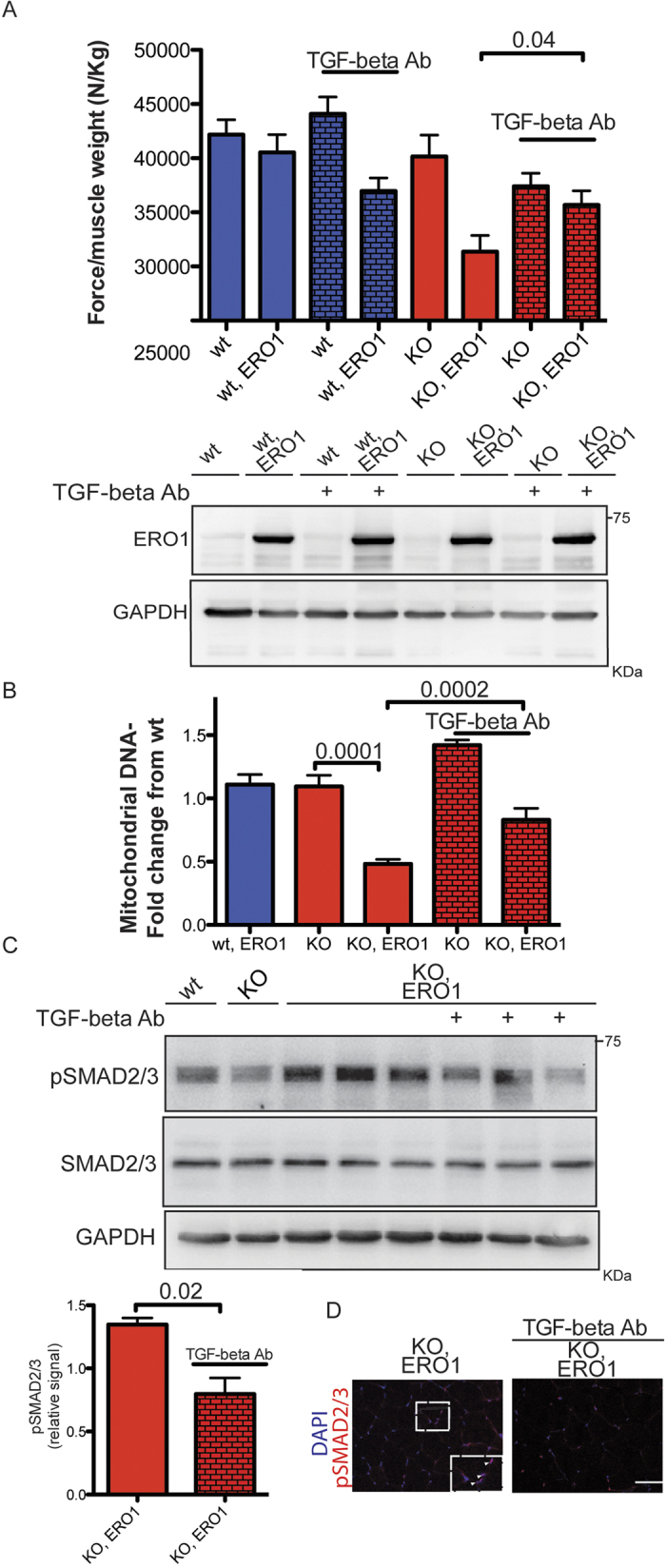
TGF-beta neutralizing antibody, by antagonizing TGF-beta, restores ERO1-injected SEPN1 KO muscle force. (**A**) Bar graphs representing the *in vivo* specific force of the gastrocnemius muscle after intraperitoneal injection of TGF-beta neutralizing antibody (stimulation frequency 100 Hz, n = 11 per group). The bar graphs indicate means ± SEM, the difference was examined using a two-tailed unpaired Student’s *t* test. P value is indicated on the graph. Below, representative ERO1α and GAPDH immunoblots of proteins extracted from the gastrocnemii of wt, wt, ERO1, KO and KO, ERO1 after TGF-beta neutralizing antibody treatment. (**B**) Real-time PCR quantification of the amount of mtDNA relative to that of RNAse-P, a nuclear gene used as a standard (n = 6 per each group). The bar graphs indicate means ± SEM, differences were examined using one-way ANOVA test and Bonferroni’s post-test. P values are indicated on the graph. (**C**) Immunoblots of pSMAD2/3 and total SMAD2/3 of proteins extracted from the gastrocnemii of wt, KO and three different KO, ERO1 and KO, ERO1 after TGF-beta neutralizing antibody treatment. GAPDH was used as loading control. Below, bar graphs showing the signal of pSMAD2/3 in KO, ERO1 and KO, ERO1 after TGF-beta neutralizing antibody treatment in relation to that of the wt (n = 3 per each group). The bar graphs show mean values ± SEM; the difference was examined using a 2-tailed unpaired Student’s *t* tests. P value is shown on the graph. (**D**) Gastrocnemii were stained for pSMAD2/3 and DAPI (scale bar: 50 μm), the inset box represents an example of muscle fibers positively stained for both pSMAD2/3 and DAPI.

As previously seen in Figs 1H and 4A shows that the normalized maximal force of the gastrocnemius muscles measured *in vivo* was lower in the *ERO1-*injected SEPN1 KO mice and accompanied by an increase in TGF-beta signalling, which is consistent with previous findings showing that treating mice with recombinant TGF-beta decreases skeletal muscle-specific strength^29^ (Fig. 4A and C). The normalised maximal muscle strength of the *ERO1-*injected SEPN1 KO mice selectively increased upon treatment with TGF-beta neutralizing antibody (Fig. 4A). Accordingly, the mtDNA, used as an indication of the mitochondria content, was markedly decreased in the gastrocnemii of ERO1-injected SEPN1 KO (compared to the WT gastrocnemii) and increased upon treatment with TGF-beta neutralizing antibody (Fig. 4B). In line with the reduced TGF-beta signalling in response to the neutralising antibody treatment, there was a general reduction in the transcripts belonging to the TGF-β pathway and in the abundance of pSMAD2/3^19^ (Figs 2B and 4C,D).

In conclusion, all of these data indicate a causal relationship between hyperactive TGF-beta signalling and the development of skeletal myopathy in SEPN1 KO mice.

### A limited concentration of Ascorbic acid in skeletal muscle unveils SEPN1-related myopathy asscociated with hyperactivity of TGF-beta signal

Unlike humans and zebrafish, in which SEPN1 loss of function gives rise to an overt muscle phenotype, SEPN1 KO mice are protected by the existence of compensatory pathways^30^.

Our findings of a faster turnover of ascorbic acid in SEPN1 KD cells may suggest that the vitamin moderates hyperoxidation and thus preserves muscle function. Accordingly, mice are still capable of producing ascorbic acid and can better cope with the lack of SEPN1, whereas human and zebrafish are ascorbic acid auxotrophs and show an overt muscle phenotype following SEPN1 deficiency^31^.

In order to test whether a limited concentration of ascorbic acid in the skeletal muscle of SEPN1 KO mice compromises muscle homeostasis and exacerbates their muscle phenotype, we used a mouse model that resembles the human condition of ascorbic acid auxotrophy.

Mice lacking L-Gulonolactone oxidase (Gulo KO mice), an enzyme in the ascorbic acid synthesis pathway, that depend on exogenous vitamin C for survival, were crossed with SEPN1 KO mice^17^,^32^. Female mice heterozygous for Gulo (Gulo Het mice) were mated with male Gulo Het mice, and female SEPN1 KO, Gulo Het mice were mated with male SEPN1 KO, Gulo Het mice, in order to recover progeny with all of the informative genotypes (wt and SEPN1 KO and Gulo KO and SEPN1 KO, Gulo KO).

**Figure 5:**
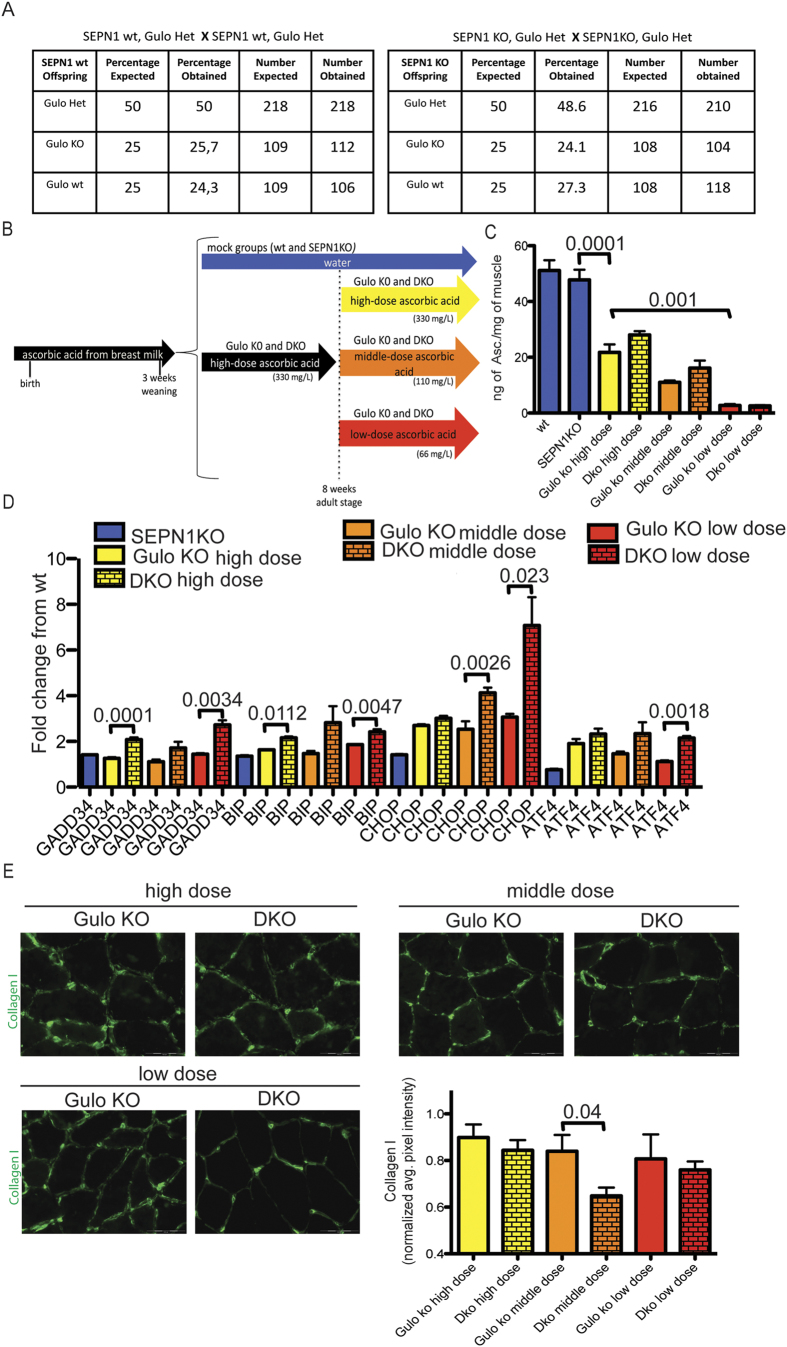
Ascorbic acid levels affect collagen deposition in the muscle of compound Gulo KO, SEPN1 KO (DKO) mice. (**A**) SEPN1 wt, Gulo Het mice were crossed with SEPN1 wt, Gulo Het mice and SEPN1 KO, Gulo Het mice were crossed with SEPN1KO, Gulo Het mice and the progeny were genotyped at P14. The expected and obtained distribution of genotypes at weaning among more then 400 mice progeny of the two crosses is indicated. The DKO (Gulo KO, SEPN1 KO) mice are recovered with the expected Mendelian frequency. (**B**) Schematic design of ascorbic acid administration. (**C**) Ascorbic acid content in gastrocnemius of the indicated genotypes of male mice maintained at the three different doses of ascorbic acid (n = 4–14 per group). The bar graphs show mean values ± SEM; the differences were examined using one-way ANOVA test and Bonferroni’s post-test. P values are indicated on the graph. (**D**) Relative abundance of UPR markers mRNAs measured by quantitative real time PCR in cDNA from gastrocnemii (n = 3 per each group). The bar graphs show mean values ± SEM; the differences were examined using a two-tailed unpaired Student’s *t* test. P values are indicated on the graph. (**E**) Representative histology of collagen I staining and relative quantification (n = 3–4 per group) (scale bar: 100 μm). The bar graphs show mean values ± SEM; the differences were examined using a two-tailed unpaired Student’s *t* test. P value is indicated on the graph.

Figure 5A shows that the mice with the double mutant genotype SEPN1 KO, Gulo KO were recovered with the frequency predicted by the Mendelian transmission of the mutant alleles, thus indicating the absence of any deleterious effect of the combined mutation on prenatal stages.

It has been reported that Gulo KO mice supplemented with ascorbic acid in water (330 mg/L) gain weight and reproduce normally^32^. Given the gender-related differences in ascorbic acid metabolism^33^, male and female SEPN1 KO and Gulo KO (DKO) mice and Gulo KO mice were separately randomised to three different dosages of ascorbic acid in water (330 mg/L [high dose], 110 mg/L [medium dose] or 66 mg/L [low dose]) (the estimated daily intake of ascorbic acid was approximately 1.6 mg, 0.5 mg and 0.3 mg for three doses respectively) at eight weeks of age, when the muscle is fully developed (Fig. 5B). The aim of this ascorbic acid titration was to find a dose that reveals the SEPN1-related muscle phenotype without inducing the more serious condition of scurvy.

The ascorbic acid content of the gastrocnemii of the treated mice was measured and was detected to be proportional to the dietary ascorbic acid supplementation (Fig. 5C and Sup. Fig. 3A). Notably, the gastrocnemii of the mice receiving the high 330 mg/L dose of ascorbic acid in their drinking water contained significantly less ascorbate than those of the WT and SEPN1KO mice, thus suggesting that this dose is sub-optimal as previously reported^34^.

A lower ascorbic acid level in muscle combined with the lack of SEPN1 increased the expression of ER stress and UPR indicators (Fig. 5D), thus suggesting a role of ascorbic acid in defending ER redox homeostasis and coping with the lack of SEPN1. Moreover, the collagen signal around muscle fibers of DKO mice maintained at the middle dose of ascorbic acid was impaired, as was the expression of HSP47, a collagen-specific molecular chaperone^35^ (Supp. Fig. 3B), thus indicating a direct relationship between muscle ascorbic acid and ECM collagen content (Fig. 5E).

However, DKO mice maintained at the low dose of ascorbic acid showed a faint collagen signal around muscle fibers but also hot spots of collagen in the space between fibers that point to a fibrotic phenotype (Sup. Fig. 3C).

**Figure 6:**
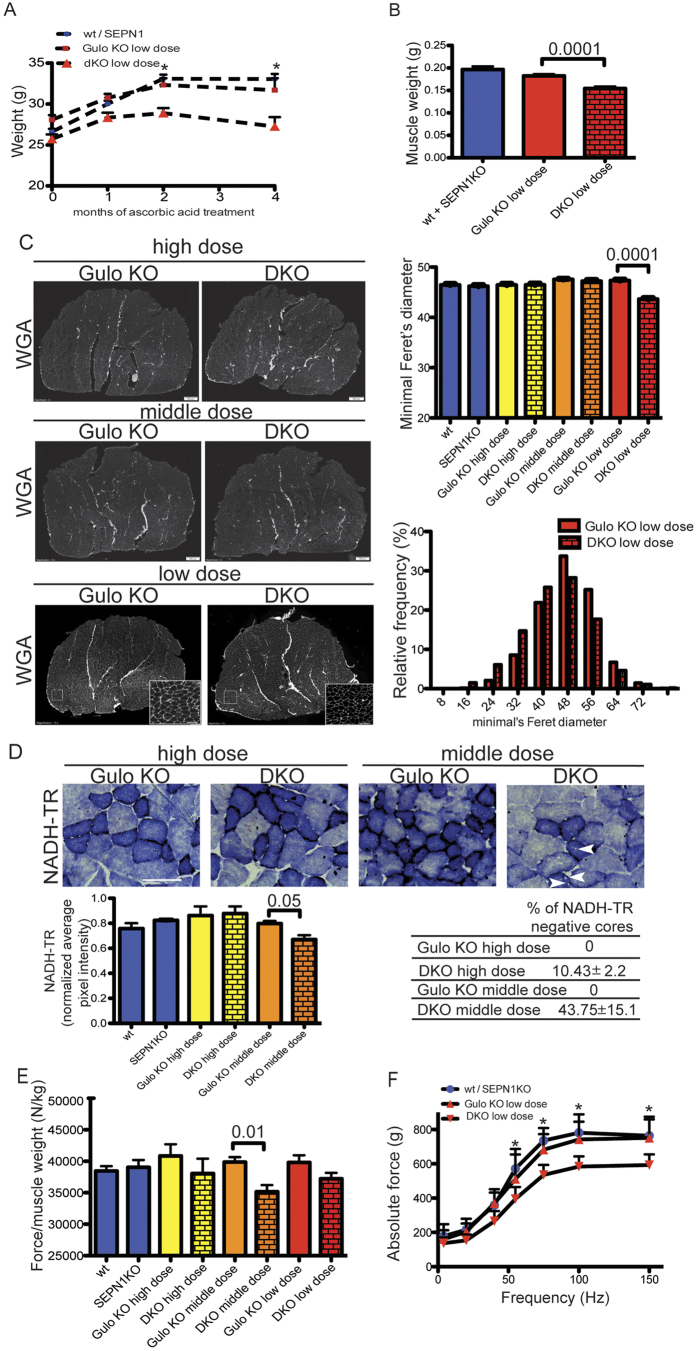
Lower Ascorbic level worsens the muscle phenotype of DKO mice. (**A**) Body weight over four months of male wt, SEPN1KO and Gulo KO, DKO mice treated with the low dose of 66 mg/L of ascorbic acid. Two way Anova followed by Bonferroni post-test was performed (n = 12 per group, *P = 0,001). (**B**) Weight of gastrocnemius at four month of wt, SEPN1 KO and Gulo KO, DKO mice treated with the low dose of 66 mg/L of ascorbic acid (n = 9/12 per group). The bar graphs indicate means ± SEM, the difference between Gulo KO and DKO was examined using a 2-tailed unpaired Student’s *t* tests. P value is shown on the graph. (**C**) Representative histology of wheat-germ agglutinin (WGA) stain and minimal Feret’s diameter (μm) of gastrocnemii (n = 4 muscles and 6000 fibers counted per condition, the bar graphs indicate means ± SEM, the difference between Gulo KO and DKO was examined using a 2-tailed unpaired Student’s *t* tests. P value is shown on the graph) (scale bar: 500 μm). Below, the relative frequency of muscle fibers of Gulo KO and DKO maintained at the low dose of ascorbic acid. (**D**) Representative histology of NADH-TR of gastrocnemii. White arrowheads indicate minicores (n = 6 per group, scale bar: 50 μm). Below, table indicating the percentage of minicore-like structures in type 1 fibers of gastrocnemii as revealed by NADH-TR labelling (average value from 100 type 1 fibers) and histological quantification of NADH-TR staining reporting for the NADH-TR activity (bar graphs indicate means ± SEM, the difference was examined using a 2-tailed unpaired Student’s *t* tests and P value is indicated on the graph). (**E**) Bar graphs representing the *in vivo* specific force of the gastrocnemii after four months of administration of ascorbic acid (n = 12 per group, the bar graphs indicate means ± SEM, the difference was examined using a 2-tailed unpaired Student’s *t* tests. P value is indicated on the graph). Please, note the diminished muscle tension of the DKO supplemented with the middle dose of ascorbic acid. (**F**) Force-frequency curve of the absolute force in male mice after four months of ascorbic acid treatment at 66 mg/L (n = 12 per group, two-way Anova followed by Bonferroni post-test was performed, *P = 0.001).

After eight weeks of age, the mice continued receiving the three doses of ascorbic acid without showing any apparent ill-health or weight loss. However, the DKO mice receiving the low dose started losing weight after three months (Fig. 6A) and, by the fourth month, had lost 10% of their original weight.

As the prominent weight loss of the DKO mice treated with the low dose of ascorbic acid indicated a selective effect on mice with the SEPN1 KO background, all of the mice were sacrificed and examined after four months. In line with their reduced body weight, the mass of the gastrocnemii of the DKO mice treated with the low dose of ascorbic acid was reduced by 25% (Fig. 6B), and quantitative analysis of the muscle fibers showed that they were the only mice with signs of atrophy that was accompanied by a lower rate of new protein synthesis (Fig. 6C) and (Fig. Sup.4 A).

However, histochemical staining for mitochondrial enzyme NADH dehydrogenase (NADH-TR) activity revealed a higher percentage of minicores, less mitochondrial DNA and less NADH-TR activity in the DKO mice treated with the medium dose of ascorbic acid than in the Gulo KO mice, thus indicating a functional defect in the mitochondria of DKO myofibers (Fig. 6D) and (Fig. Sup. 4B).

The normalized maximal strength of the gastrocnemius muscle measured *in vivo* tended to be less in the DKO mice regardless of the ascorbic acid dose, but was significantly less only in those treated with the medium dose (Fig. 6E). However the muscle atrophy observed in the DKO mice treated with the lowest dose of ascorbic acid was associated with reduced absolute muscle strength (Fig. 6F).

**Figure 7:**
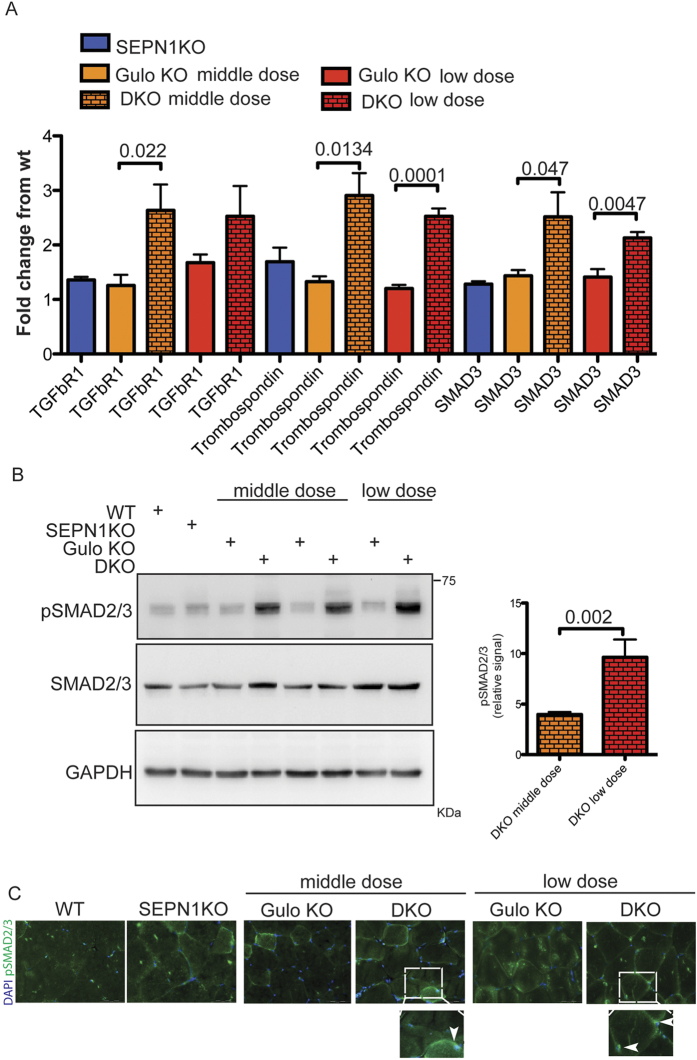
The muscle phenotype in DKO is triggered by an hyperactive TGF-beta. (**A**) Relative abundance of three TGF-beta pathway mRNAs measured using quantitative real-time PCR in cDNA from gastrocnemii (n = 5 per group). The bar graphs indicate means ± SEM, P values are indicated on the graph. (**B**) Immunoblots of pSMAD2/3 and total SMAD2/3 proteins extracted from gastrocnemii. GAPDH was used as the loading control. The bar graphs on the right show the pSMAD2/3 signal in DKO muscle from the mice fed the medium and low dose of ascorbic acid in relation to that of the muscle of Gulo KO mice fed the same doses of ascorbic acid (n = 3 per group). The bar graphs show mean values ± SEM; the differences were examined using a 2-tailed unpaired Student’s *t* test. P value is shown on the graphs. (**C**) Gastrocnemii were stained for pSMAD2/3 and DAPI (scale bar: 100 μm), the inset box represents an example of muscle fibers positively stained for both pSMAD2/3 and DAPI (n = 3–4 muscle per each group).

The muscle defect in the DKO mice treated with the two lower doses of ascorbic acid was accompanied by higher levels of transcripts belonging to the TGF-beta pathway (Fig. 7A) and a stronger pSMAD2/3 signal (Fig. 7B,C), thus indicating that the hyperactivity of the TGF-beta signal was triggered by the lower concentrations of ascorbic acid.

In conclusion, a medium dose of ascorbic acid impairs DKO muscle fibers and the production of muscle strength, whereas a lower dose, accompanied by an higher TGF-beta activity, triggers the more serious muscle atrophy, thus leading to smaller muscle fibers.

## Discussion

Loss-of-function mutations in the human *SEPN1* gene are involved in early-onset recessive neuromuscular disorders and, although the function of SEPN1 has not yet been clearly characterised, it is thought that dysregulated calcium homeostasis and oxidative stress contribute to the pathogenesis of SEPN1-related myopathies. This study of SEPN1 KO mice as a model of ER hyperoxidation provides new mechanistic insights into the chain of events connecting oxidative stress and myopathies, and may therefore contribute to the more effective treatment of SEPN1-related myopathies and, possibly, many other muscle disorders associated with oxidative stress.

We have recently shown that SEPN1 redox regulates SERCA, a pump responsible for Ca^2+^ reuptake into the sarcoplasmic reticulum during excitation contraction (EC) coupling, and copes with the potentially harmful effects of ERO1, the main disulfide oxidase of the ER, whose expression is regulated by the UPR^16^.

As SEPN1 KO mice are somehow protected from the effects of SEPN1 loss^17^,^18^, we delivered the disulfide oxidase ERO1 into the gastrocnemius muscle, where its expression forced the ER to be hyperoxidised, as previously shown by the higher ratio between oxidised and total glutathione, triggering ER stress^16^ and (Fig. 1). In line with the two-hit hypothesis underlying the pathogenetic mechanism of muscle dystrophies, the ERO1 surge acts as a second hit in SEPN1KO muscle to reveal a SEPN1-related myopathy, whereas WT muscles are protected^36^ and (Fig. 1). When the levels of ERO1 surges in SEPN1KO mice, muscle minicores become apparent and muscle force normalised for muscle weight is significantly reduced at all stimulation frequencies (Fig. 1), thus indicating that the over-expression of ERO1 in SEPN1KO muscle creates a dysfunction that is similar to that observed in minicore myopathies.

We therefore used the SEPN1 KO/ERO1-induced muscle model to investigate how ERO1 and consequent ER hyperoxidation trigger muscle pathology. ERO1 activity in mammals is normally counteracted by ER peroxidases such as GPX7, GPX8 and PRDX4^37^,^38^,^39^, so we explored whether the muscle phenotype due to an excess of ERO1 was not only the consequence of direct ERO1-mediated hyperoxidation of proteins but was also indirect, through the activation of pathways important for skeletal muscle. Microarray studies of SEPN1 KO/ERO1-injected muscle indicated a strong TGF-beta signature. This is an interesting finding as the TGF-beta pathway has been extensively studied in skeletal muscle because of its involvement in many forms of myopathic disorders (Fig. 2).

The results of our SEPN1 KD myoblast experiments indicate a faster turnover of the reductant (anti-oxidant) ascorbic acid, which hyperactivated TGF-beta by impairing collagen biogenesis and deposition in the ECM (Fig. 3). Interestingly, unlike SEPN1 KO muscle, SEPN1 KD myoblasts without ERO1 over-expression show a high level of TGF-beta activity and a phenotype compatible with TGF-beta hyperactivation. What may account for this difference in the outcome of SEPN1 loss-of-function in the two systems of skeletal muscle and myoblasts is the scurvy-like condition in which the cells were cultured, and the fact that the cultured cells were exposed to higher oxygen concentrations than skeletal muscle, which induced them to activate the TGF-beta pathway. These observations once again indicate that oxidative stress is an important pathophysiological mechanism in SEPN1 loss-of-function, and that ascorbic acid can cope with the consequences of ER hyperoxidation as a general reductant.

We therefore used Gulo KO mice unable to synthesise ascorbic acid and SEPN1 KO mice that are prone to developing myopathy in order to investigate whether reduced dietary levels of ascorbic acid affect muscle homeostasis. Our findings demonstrate that, by inducing ER stress and affecting TGF-beta activity (Figs 5 and 7), a deficiency of vitamin C induces a SEPN1-related pathological muscle phenotype in SEPN1 KO mice, whose severity is proportional to the muscle concentration of the vitamin and hyperactivity of the TGF-beta pathway: a medium dose of ascorbic acid impairs muscle fibers and the production of muscle strength, whereas a lower dose triggers more serious muscle atrophy, thus leading to smaller muscle fibers and fibrosis (Fig. 6). This progressive worsening of the muscle phenotype proceeds at the same pace as the muscle oxidative poise imposed by the reduced level of ascorbic acid, which proportionally influences TGF-beta activity. This may help to explain the heterogeneity of the muscle phenotype in patients with SEPN1-related myopathy, which may be due to different levels of muscle oxidative poise that influence TGF-beta activity^40^.

As shown by ERO1*-*injected SEPN1 KO mice and the compound DKO mice fed on the medium dose of ascorbic acid, the pathological cause of SEPN1-related myopathies is not primarily due to the TGF-beta-dependent fibrosis (only seen in DKO mice fed on the low dose), but to a TGF-beta-dependent functional defect in muscle fibers that affects muscle strength. Indeed, TGF-beta hyperactivity may lead to muscle weakness by decreasing the production of ER calcium-induced muscle strength^41^. Future investigations should therefore be aimed at showing whether the pathological cause of this defect is related to the levels of ER calcium, which are worsened by TGF-beta hyperactivity.

Although no data are available from patients affected by SEPN1-related myopathies that indicate hyperactivation of the TGF-beta pathway, such hyperactivation is consistent with the muscle fibrosis encountered in some of them^42^. As our data suggest that systemic antagonism of TGF-beta re-established skeletal muscle function in SEPN1 mutant mice (Fig. 4), further studies should try to clarify whether interfering with the TGF-beta pathway can be curative in patients affected by SEPN1-related myopathies.

Theoretically, as the skeletal muscle ER is engaged in stable structural and functional interactions with mitochondria (MAMs), ER hyperoxidation may be secondary to that of the juxtaposed mitochondria, and so it is worth investigating whether the pathological mechanism described above is common to the mitochondrial myopathies frequently associated with oxidative stress^13^. As some myopathies are associated with oxidative stress but not with fibrosis, it is tempting to speculate that targeting the TGF-beta/ERO1/ascorbic acid axis may also represent a new therapeutic approach to hyperoxidation-induced myopathies accompanied by an absence of fibrosis.

Finally, decreased tissue ascorbic acid levels associated with a strong TGF-beta signature and a conspicuous skeletal muscle phenotype has also been found in another mouse model of ER hyperoxidation, thus suggesting that we have learned something fundamental about muscle biology: ER hyperoxidation triggers the TGF-beta pathway, and this gives rise to a skeletal muscle phenotype by perturbing the homeostasis of muscle fibres (Zito. E, unpublished results) and^22^. It still has to be determined whether TGF-beta hyperactivation is a consequence of the UPR, or whether the pathways of TGF-beta and UPR only have the common trigger of ER hyperoxidation, and then proceed in parallel without influencing each other. The TGF-beta response to the attenuation of the CHOP/ERO1 pathway in SEPN1-related myopathic muscles will help to clarify this point.

## Materials and Methods

### Transgenic mice and treatments

All procedures involving animals and their care carried out at the Mario Negri Institute and University of Padua were conducted as described by the institutional guidelines that are in accordance with national (D.L. no. 116, G.U. suppl. 40, Feb. 18, 1992, No.8, G.U., 14 luglio 1994) and international laws and policies (EEC Council Directive 86/609, OJ L 358, 1DEC.12,1987; NIH Guide for the Care and use of Laboratory Animals, U.S. National Research Council, 1996). The SEPN1KO mice were purchased from the EMMA repository (Sepn1 < tm1.2Mred > /Orl). Genotyping at the *SepN1* locus followed published procedures^17^. The Gulo KO mice were purchased from the MMRC repository. Genotyping at the *Gulonololactone L oxidase* locus followed published procedures^32^. For analysis of the rate of protein synthesis, we used the SUnSET method^43^: the animals were starved 30 min, injected with puromycin (0.040 μmol/g puromycin), and sacrificed 30 min after injection. In order to induce fibrosis, 10 μg of cardiotoxin γ (Latoxan, Valence, France) was injected into the gastrocnemius of 2-month-old mice, and their contralateral limbs were sham-injected with PBS. The mice were euthanised 10 days after injection, and the muscles were harvested.

### Histological and electromicroscopic analysis of muscles

Muscles were flash-frozen in isopentane precooled in liquid nitrogen, embedded in optimal cutting temperature compound, and cross sections (8 μm thick) were cut with a cryostat. Cryosections were processed for nicotinamide adenine dinucleotide-TR (NADH-TR) staining or modified Gomori’s Trichrome. For immunofluorescence analysis, muscle cryosections were incubated with a solution of PBS containing 10% bovine serum albumin (BSA) and mounted with aqueous mounting medium (Fluorescence Mounting Medium, Dako). Muscle section were acquired at 10X by Olympus BX-61 Virtual Stage microscope.

The fiber size diameter (minimal Feret’s diameter) were determined on frozen muscle sections stained with WGA.

For ultrastructural studies, samples were fixed with 2.5% glutaraldehyde in 10 mM phosphate buffer (pH 7.4).

### *In vivo* muscle mechanics

*In vivo* gastrocnemius muscle contractile performance was measured using a 305B muscle lever system (Aurora Scientific Inc.) in mice anesthetized with a mixture of Xylotine and Zoletil. Mice were placed on a thermostatically controlled table, the knee was kept stationary and the foot was firmly fixed to a footplate, which was connected to the shaft of the motor. Contraction was elicited by electrical stimulation of the sciatic nerve. Teflon-coated 7 multistranded steel wires (AS 632, Cooner Sales, Chatsworth, CA, U.S.A.) were implanted with sutures on either side of the sciatic nerve proximally to the knee before its branching. At the distal ends of the two wires the insulation was removed, while the proximal ends were connected to a stimulator (Grass S88). In order to avoid recruitment of the dorsal flexor muscles, the common peroneal nerve was cut. The torque developed during isometric contractions was measured at stepwise increasing stimulation frequency, with pauses of at least 30 seconds between stimuli to avoid effects due to fatigue. Duration of the trains never exceeded 600 ms. Force developed by plantar flexor muscles was calculated by dividing torque by the lever arm length (taken as 2.1 mm).

### Microarray analysis

The microarray data discussed in this publication have been deposited in the ArrayExpress Archive of Functional Genomics Data hosted by the European Bioinformatics Institute (EMBL-EBI, ebi.ac.uk) and are accessible through the accession no. E-MTAB-4933. Total RNA was isolated from muscle tissues using the RNeasy Mini Kit (Qiagen) according to the manufacturer’s instructions. Genechip Mouse Gene 2.1 ST arrays (Affymetrix) were used for whole genome gene-expression analysis of biological duplicated samples derived from WT and SEPN1 KO mice in presence of either ERO1 or PBS. RNA to probe hybridization was performed by the IFOM microarray core facility. Data were pre-processed in *R* statistical environment using the *oligo* package and normalized using the robust multi-array average (RMA) algorithm. Probe-level summarized intensities were used for down-stream analysis. Two-way analysis of variance (*ANOVA*) was used to compute statistical significance (P < 0.01) between experimental conditions (genotype/treatment) and their interaction. Pathway analyses were performed using the web based gene-set analysis toolkit (WebGestalt – bioinfo.vanderbilt.edu) which performs a hypergeometric test for the enrichment of GO terms and pathways, followed by the Benjamini & Hochberg method for multiple test adjustment.

### Antibodies

We used the following antibodies for Western blotting: rabbit anti-SMAD2/3p and mouse anti-Actin from Santa Cruz, rabbit anti-SMAD2/3 from abcam, mouse anti-GAPDH and rabbit anti-SEPN1 from Sigma, rabbit anti-collagen I from Rockland, mouse anti-puromycin from Merck Millipore, rabbit anti-ERO1α^44^, wheat germ agglutinin Alexa fluor 488 coniugate from Molecular Probes. TGF-beta Pan specific antibody from R&D systems was used at 1 mg/kg to inject mice.

### 4-Hydroxyproline and soluble collagen content

4-Hydroxyproline content of cell acid hydrolysate was measured using the QuickZyme Total collagen assay kit (QuickZyme BioSciences, Netherlands) and the secretion of soluble collagen was measured in the three days conditioned media of C2C12 cells using the Soluble collagen assay kit (QuickZyme BioSciences, Netherlands) according to the manufacturer’s instruction.

### TGF-beta bioassay

To quantify the levels of active and total TGF-beta, we used mink lung epithelial cells (MLEC) that produce luciferase under the control of the PAI-1 promoter in response to TGF-beta (kind gift from Daniel Rifkin, New York University)^45^. Luciferase activity was measured with a luciferase assay system kit (Promega). PAI-1 (plasminogen activator inhibitor promoter)/luciferase construct-transfected MLECs were incubated with conditioned media coming from WT and KD cells or various concentration of human recombinant TGF-beta 1 at 37 °C for 20 h. There was a strict dose-dependent increase in luciferase activity (RLU) by TGF-β1 between 0 and 0.1 ng/mL.

### Ascorbic acid content in cells

Ascorbic acid in the cell and muscle was extracted by a solution composed by 60% methanol and 1 mM EDTA then centrifuged to pellet the protein (10,600 *g* for 5 min).

Ascorbic acid in cells was analyzed by using reverse-phase HPLC with a Synergi 4 micron Fusion-RP 80 A column (Phenomenex) and coulometric detection. The chromatograph consisted of Shimadzu LC-10AD pump (Shimadzu, Italy), refrigerated (4 °C) 717 autosampler (Waters, Italy), Coulochem II electrochemical detector equipped with a 5014B electrochemical cell consisting of two in-series electrodes (ESA, Chelmsford, MA) and software for acquisition and calculation of chromatographic data (Azur 5.0; Datalys, France). Detector settings were electrode 2.200 mV; electrode 1.0 mV. Mobile phase contained 50 mM sodium phosphate monobasic, 50 mM sodium acetate anhydrous, 0.5 mM acetyltrimethylammonium bromide dissolved in HPLC-grade water (pH 4.8). Methanol percentage was adjusted to 20% of final volume and the mobile phase was filtered through 0, 45 μm membrane filter (Millipore) and degassed. The column was conditioned with mobile phase at 1 mL/min for 36–48 h before running standards and samples and washed after every 30–50 biological samples with 50% methanol/water for 12 hours at 1 mL/min.

Oxidation potentials setting for the electrochemical cell were E1 0 mV; E2 + 200 mV.

The assay was calibrated daily with fresh solutions (2.5–40 ng of standard ascorbic acid) diluted in the mobile phase. Retention time of ascorbic acid was 6.05 min.

### Ascorbic acid content in muscle

The column was 4 mm particle size, 150 × 4.6 mm, Accucore X C18 (Thermo Scientific, Italy) protected with a drop-in 10 × 4.6 mm guard column (Thermo Scientific, Italy). The mobile phase consisted of phosphate-acetate buffer prepared by dissolving 50 mM NaH_2_PO_4_ and 50 mM CH_3_COONa in about 750 mL ultrapure water (Milli-Q; Millipore, Italy). Two-hundred mL methanol containing 0.5 mM N-cetyl-N,N,N, trymethylammonium bromide (CTMAB) were added to the buffer solution, the pH of the solution adjusted to 5.0 with concentrated H_3_PO_4_ and the volume brought to 1 L with ultrapure water. After filtering through 0.45 mm regenerated cellulose membrane filters (Sartorius Stedim Biotech, Germany),and degassing under vacuum, the mobile phase was pumped through the HPLC system at a constant flow rate of 1 mL/min. The column and guard column were conditioned with mobile phase at 1 mL/min 48 h before running standards and samples.

Oxidation potentials setting for the electrochemical cell were E1 0 mV; E2 + 200 mV. Ascorbic acid was read as the second electrode output signal. The assay was calibrated daily with fresh solutions (2.5–40 ng of standard ascorbic acid) diluted in the mobile phase. Retention time of ascorbic acid was 3.20 min.

### Statistics

Statistical analysis was performed using GraphPad Prism. All results are presented as mean ± standard error of the mean (SEM). Differences were examined using a 2-tailed unpaired Student’s *t* tests and in the case of small sample size the Welch’s correction was applied (Figs 1B, 3C,D, 5D, 6C and 7A). In the case of multiple comparisons one-way or two-way ANOVA test and Bonferroni’s post-test was used.

### Study approval

All of the procedures on mice were reviewed and approved by the Comitato etico dell’Istituto di Ricerche Farmacologiche Mario Negri and the University of Padua (Italy).

## Additional Information

**How to cite this article**: Pozzer, D. *et al*. Endoplasmic Reticulum Oxidative Stress Triggers Tgf-Beta-Dependent Muscle Dysfunction by Accelerating Ascorbic Acid Turnover. *Sci. Rep.* **7**, 40993; doi: 10.1038/srep40993 (2017).

**Publisher's note:** Springer Nature remains neutral with regard to jurisdictional claims in published maps and institutional affiliations.

## Supplementary Material

Supplementary Information

## References

[b1] Vembar S. S. & Brodsky J. L. One step at a time: endoplasmic reticulum-associated degradation. Nat Rev Mol Cell Biol 9(12), 944–957 (2008). 19002207 10.1038/nrm2546 PMC2654601

[b2] Iwawaki T., Akai R., Kohno K. & Miura M. A transgenic mouse model for monitoring endoplasmic reticulum stress. Nat Med 10(1), 98–102 (2004). 14702639 10.1038/nm970

[b3] Moorwood C. & Barton E. R. Caspase-12 ablation preserves muscle function in the mdx mouse. Hum Mol Genet 23(20), 5325–5341 (2014). 24879640 10.1093/hmg/ddu249 PMC4168821

[b4] Lin Y. Y. . Zebrafish Fukutin family proteins link the unfolded protein response with dystroglycanopathies. Hum Mol Genet 20(9), 1763–1775 (2011). 21317159 10.1093/hmg/ddr059 PMC3071672

[b5] Demontis F., Piccirillo R., Goldberg A. L. & Perrimon N. Mechanisms of skeletal muscle aging: insights from Drosophila and mammalian models. Dis Model Mech 6(6), 1339–1352 (2013). 24092876 10.1242/dmm.012559 PMC3820258

[b6] Vitadello M., Doria A., Tarricone E., Ghirardello A. & Gorza L. Myofiber stress-response in myositis: parallel investigations on patients and experimental animal models of muscle regeneration and systemic inflammation. Arthritis Res Ther 12(2), R52 (2010). 20334640 10.1186/ar2963 PMC2888201

[b7] Ron D. & Walter P. Signal integration in the endoplasmic reticulum unfolded protein response. Nat Rev Mol Cell Biol 8(7), 519–529 (2007). 17565364 10.1038/nrm2199

[b8] Marciniak S. J. . CHOP induces death by promoting protein synthesis and oxidation in the stressed endoplasmic reticulum. Genes Dev 18(24), 3066–3077 (2004). 15601821 10.1101/gad.1250704 PMC535917

[b9] Tu B. P. & Weissman J. S. The FAD- and O(2)-dependent reaction cycle of Ero1-mediated oxidative protein folding in the endoplasmic reticulum. Mol Cell 10(5), 983–994 (2002). 12453408 10.1016/s1097-2765(02)00696-2

[b10] Gross E., Kastner D. B., Kaiser C. A. & Fass D. Structure of Ero1p, source of disulfide bonds for oxidative protein folding in the cell. Cell 117(5), 601–610 (2004). 15163408 10.1016/s0092-8674(04)00418-0

[b11] Harding H. P. . An integrated stress response regulates amino acid metabolism and resistance to oxidative stress. Mol Cell 11(3), 619–633 (2003). 12667446 10.1016/s1097-2765(03)00105-9

[b12] Zito E. ERO1: A protein disulfide oxidase and H2O2 producer. Free Radic Biol Med 83, 299–304 (2015). 25651816 10.1016/j.freeradbiomed.2015.01.011

[b13] Eisner V., Csordas G. & Hajnoczky G. Interactions between sarco-endoplasmic reticulum and mitochondria in cardiac and skeletal muscle - pivotal roles in Ca(2)( + ) and reactive oxygen species signaling. J Cell Sci 126(Pt 14), 2965–2978 (2013). 23843617 10.1242/jcs.093609 PMC3711195

[b14] Balaban R. S., Nemoto S. & Finkel T. Mitochondria, oxidants, and aging. Cell 120(4), 483–495 (2005). 15734681 10.1016/j.cell.2005.02.001

[b15] Lavery G. G. . Deletion of hexose-6-phosphate dehydrogenase activates the unfolded protein response pathway and induces skeletal myopathy. J Biol Chem 283(13), 8453–8461 (2008). 18222920 10.1074/jbc.M710067200 PMC2417187

[b16] Marino M. . SEPN1, an endoplasmic reticulum-localized selenoprotein linked to skeletal muscle pathology, counteracts hyper-oxidation by means of redox-regulating SERCA2 pump activity. Hum Mol Genet (24), 1843–1855 (2015). 25452428 10.1093/hmg/ddu602

[b17] Rederstorff M. . Increased muscle stress-sensitivity induced by selenoprotein N inactivation in mouse: a mammalian model for SEPN1-related myopathy. PLoS One 6(8), e23094 (2011). 21858002 10.1371/journal.pone.0023094 PMC3152547

[b18] Moghadaszadeh B. . Selenoprotein N deficiency in mice is associated with abnormal lung development. FASEB J 27(4), 1585–1599 (2013). 23325319 10.1096/fj.12-212688 PMC3606527

[b19] Cohn R. D. . Angiotensin II type 1 receptor blockade attenuates TGF-beta-induced failure of muscle regeneration in multiple myopathic states. Nat Med 13(2), 204–210 (2007). 17237794 10.1038/nm1536 PMC3138130

[b20] Heldin C. H., Miyazono K. & ten Dijke P. TGF-beta signalling from cell membrane to nucleus through SMAD proteins. Nature 390(6659), 465–471 (1997). 9393997 10.1038/37284

[b21] Ramirez F. & Rifkin D. B. Extracellular microfibrils: contextual platforms for TGFbeta and BMP signaling. Curr Opin Cell Biol 21(5), 616–622 (2009). 19525102 10.1016/j.ceb.2009.05.005 PMC2767232

[b22] Zito E., Hansen H. G., Yeo G. S., Fujii J. & Ron D. Endoplasmic Reticulum Thiol Oxidase Deficiency Leads to Ascorbic Acid Depletion and Noncanonical Scurvy in Mice. Mol Cell (2012). 10.1016/j.molcel.2012.08.010 PMC3473360 22981861

[b23] Lamande S. R. & Bateman J. F. Procollagen folding and assembly: the role of endoplasmic reticulum enzymes and molecular chaperones. Semin Cell Dev Biol 10(5), 455–464 (1999). 10597628 10.1006/scdb.1999.0317

[b24] Juva K., Prockop D. J., Cooper G. W. & Lash J. W. Hydroxylation of proline and the intracellular accumulation of a polypeptide precursor of collagen. Science 152(3718), 92–94 (1966). 5910019 10.1126/science.152.3718.92

[b25] May J. M. Vitamin C transport and its role in the central nervous system. Subcell Biochem 56, 85–103 (2012). 22116696 10.1007/978-94-007-2199-9_6 PMC3725125

[b26] Zito E. PRDX4, an ER-localised peroxiredoxin at the crossroads between enzymatic oxidative protein folding and non-enzymatic protein oxidation. Antioxid Redox Signal (2012). 10.1089/ars.2012.4966 23025503

[b27] Monteiro G., Horta B. B., Pimenta D. C., Augusto O. & Netto L. E. Reduction of 1-Cys peroxiredoxins by ascorbate changes the thiol-specific antioxidant paradigm, revealing another function of vitamin C. Proc Natl Acad Sci USA 104(12), 4886–4891 (2007). 17360337 10.1073/pnas.0700481104 PMC1829234

[b28] Van Duijn M. M., Van der Zee J., VanSteveninck J. & Van den Broek P. J. Ascorbate stimulates ferricyanide reduction in HL-60 cells through a mechanism distinct from the NADH-dependent plasma membrane reductase. J Biol Chem 273(22), 13415–13420 (1998). 9593673 10.1074/jbc.273.22.13415

[b29] Mendias C. L. . Transforming growth factor-beta induces skeletal muscle atrophy and fibrosis through the induction of atrogin-1 and scleraxis. Muscle Nerve 45(1), 55–59 (2012). 22190307 10.1002/mus.22232 PMC3245632

[b30] Jurynec M. J. . Selenoprotein N is required for ryanodine receptor calcium release channel activity in human and zebrafish muscle. Proc Natl Acad Sci USA 105(34), 12485–12490 (2008). 18713863 10.1073/pnas.0806015105 PMC2527938

[b31] Linster C. L. & Van Schaftingen E. Vitamin C. Biosynthesis, recycling and degradation in mammals. FEBS J 274(1), 1–22 (2007). 10.1111/j.1742-4658.2006.05607.x 17222174

[b32] Maeda N. . Aortic wall damage in mice unable to synthesize ascorbic acid. Proc Natl Acad Sci USA 97(2), 841–846 (2000). 10639167 10.1073/pnas.97.2.841 PMC15418

[b33] Nakata Y. & Maeda N. Vulnerable atherosclerotic plaque morphology in apolipoprotein E-deficient mice unable to make ascorbic Acid. Circulation 105(12), 1485–1490 (2002). 11914259 10.1161/01.cir.0000012142.69612.25

[b34] Vissers M. C., Bozonet S. M., Pearson J. F. & Braithwaite L. J. Dietary ascorbate intake affects steady state tissue concentrations in vitamin C-deficient mice: tissue deficiency after suboptimal intake and superior bioavailability from a food source (kiwifruit). Am J Clin Nutr 93(2), 292–301 (2010). 21123463 10.3945/ajcn.110.004853

[b35] Ishida Y. & Nagata K. Hsp47 as a collagen-specific molecular chaperone. Methods Enzymol 499, 167–182 (2011). 21683254 10.1016/B978-0-12-386471-0.00009-2

[b36] Rando T. A. Role of nitric oxide in the pathogenesis of muscular dystrophies: a “two hit” hypothesis of the cause of muscle necrosis. Microsc Res Tech 55(4), 223–235 (2001). 11748861 10.1002/jemt.1172

[b37] Zito E. . Oxidative protein folding by an endoplasmic reticulum-localized peroxiredoxin. Mol Cell 40(5), 787–797 (2010). 21145486 10.1016/j.molcel.2010.11.010 PMC3026605

[b38] Ramming T., Hansen H. G., Nagata K., Ellgaard L. & Appenzeller-Herzog C. GPx8 peroxidase prevents leakage of H2O2 from the endoplasmic reticulum. Free Radic Biol Med 70, 106–116 (2014). 24566470 10.1016/j.freeradbiomed.2014.01.018

[b39] Tavender T. J., Springate J. J. & Bulleid N. J. Recycling of peroxiredoxin IV provides a novel pathway for disulphide formation in the endoplasmic reticulum. EMBO J 29(24), 4185–4197 (2010). 21057456 10.1038/emboj.2010.273 PMC3018787

[b40] Ferreiro A. . Mutations of the selenoprotein N gene, which is implicated in rigid spine muscular dystrophy, cause the classical phenotype of multiminicore disease: reassessing the nosology of early-onset myopathies. Am J Hum Genet 71(4), 739–749 (2002). 12192640 10.1086/342719 PMC378532

[b41] Waning D. L. . Excess TGF-beta mediates muscle weakness associated with bone metastases in mice. Nat Med 21(11), 1262–1271 (2015). 26457758 10.1038/nm.3961 PMC4636436

[b42] Bonnemann C. G. . Diagnostic approach to the congenital muscular dystrophies. Neuromuscul Disord 24(4), 289–311 (2014). 24581957 10.1016/j.nmd.2013.12.011 PMC5258110

[b43] Schmidt E. K., Clavarino G., Ceppi M. & Pierre P. SUnSET, a nonradioactive method to monitor protein synthesis. Nat Methods 6(4), 275–277 (2009). 19305406 10.1038/nmeth.1314

[b44] Zito E., Chin K. T., Blais J., Harding H. P. & Ron D. ERO1-beta, a pancreas-specific disulfide oxidase, promotes insulin biogenesis and glucose homeostasis. J Cell Biol 188(6), 821–832 (2010). 20308425 10.1083/jcb.200911086 PMC2845084

[b45] Abe M. . An assay for transforming growth factor-beta using cells transfected with a plasminogen activator inhibitor-1 promoter-luciferase construct. Anal Biochem 216(2), 276–284 (1994). 8179182 10.1006/abio.1994.1042

