# Supplementary material for: Endoplasmic Reticulum Oxidative Stress Triggers Tgf-Beta-Dependent Muscle Dysfunction by Accelerating Ascorbic Acid Turnover
Source: Sci Rep. 2017 Jan 20;7:40993. doi: 10.1038/srep40993 (PMC5247721; doi:10.1038/srep40993)

ENDOPLASMIC RETICULUM OXIDATIVE STRESS TRIGGERS TGF-BETA-DEPENDENT MUSCLE DYSFUNCTION BY ACCELERATING ASCORBIC ACID TURNOVER

Diego Pozzer[1], Mariagrazia Favellato[1], Marco Bolis[1], Roberto William Invernizzi[1], Francesca Solagna[2], Bert Blaauw[2,3], Ester Zito[4*]

Material and Methods

Cell culture

C2C12 cells were cultured in DMEM supplemented with 25 mM glucose and 10% fetal calf serum (FCS). SEPN1 was knocked down as described in [1].

Western blot analysis

Muscle protein lysates were prepared after the mechanic pulverization and homogenization in lysis buffer. Whole cell protein lysates and muscle protein lysates were prepared with RIPA lysis buffer containing 10mM Tris-HCL pH7.4, 30mM NaCl, 1mM EDTA, 1% Nonidet P-40 and protease inhibitors. For the detection of unfolded procollagen the NP-40 solubilized lysates was challenged with 0.5% SDS and centrifugated through a glycerol cushion. For the detection of puromycin, muscle protein lysates were prepared with 40mM Tris PH 7.5, 1mM EDTA, 5mM EGTA pH8.5 and 0.5% Triton and protease and phosphatase inhibitors. Protein concentration was determined for each sample by using the Bicinchoninic acid assay and equal amounts of proteins were loaded and resolved by 10% SDS-PAGE. Full list of antibodies used is provided in the paragraph Antibodies.

AAV injection

ERO1 and GFP-AAV2/1 vectors were produced by the TIGEM AAV Vector Core by triple transfection of HEK-293 cells followed by two rounds of $CsCl_2$ purification. For each viral preparation, physical titers (genome copies-GC/ml) were determined by averaging the titer achieved by dot-blot analysis and by PCR quantification using TaqMan (Applied Biosystems, Carlsbad, CA, USA).

One-month-old WT and SEPN1KO mice were anesthetized and injected with a total dose of 10^{11} GC of *ERO1*a-AAV2/1 in three sites of the right gastrocnemius. Equivalent doses of GFP-AAV2/1 or equal volumes of PBS were injected into the contralateral muscle. The animals were sacrificed 4 weeks after being injected and gastrocnemii, free from neighbouring muscles were isolated and analysed.

Real-time quantitative RT-PCR analysis

Total RNA was isolated from muscle tissues using the RNeasy Mini Kit (Qiagen) according to the manufacturer's instructions. One μg of total RNA was reverse-transcribed and analyzed with the Applied Biosystems Real-time PCR System using the ΔΔCt method. Relative gene expression was normalized to *GAPDH* mRNA levels (as was the houskeeping gene with a lower coefficient of variance among the microarray data) and the graphs show the mean expression levels of the indicated genes in the muscles of the indicated genotipes relative to those of the WT muscles.

The Real time primers were as follows:

GADD34 F: GAGGGACGCCCACAACTTC

R: TTACCAGAGACAGGGGTAGGT

CHOP F: CCACCACACCTGAAAGCAGAA

R: AGGTGAAAGGCAGGGACTCA

BIP F: TCATCGGACGCACTTGGAA

R: CAACCACCTTG AATGGCAAGA

ATF4F:ATGGCCGGCTATGGATGAT

R:CGAAGTCAAACTCTTTCAGATCCATT

TGFbeta R1 F: GCTCGACGCTGTTCTATTGG

R:CTCTCAAGGCCTCACAGCTC

Thrombospondin F:CTGCCAGAACTCGGTTACCA

R:TCCACTCAGACCAGGGAGAC

TGFbeta IF1 F: TGGACGGTGCTTGCTTTAAC

R: GGAATGGCCCTCACAACCTT

SMAD3 F: AAGAAGCTCAAGAAGACGGGG

R: ACAGGCGGCAGTAGATAACG

Collagen I F: GCGTGAAAGGACACAGTGGT

R: TTCCATTCTCTCCAGGGGCA

Collagen III F: TGGTGAACCTGGTCAAGCTG

R: TGGGCCTTTGATACCTGGAG

Collagen VI F: TTCCAAGACTGCCCTGTGGA

R: CGCATTCCAAACCAGGTTGC

HSP47 F: GAAGGCTGTCGCCATCTC

R: TCCTGCCAGATGTTTCTGC

GAPDH F: CCTTCATTGACCTCAACTAC

R: GGAAGGCCATGCCAGTGAGC

mtDNA Copy Number Analysis

Total DNA was extracted from gastrocnemii following standard procedures. MtDNA copy number, relative to the diploid chromosomal DNA content, was quantitatively analyzed in gastrocnemii using the Applied Biosystems Real-time PCR System. Primers were designed within cytochrome c oxidase subunit 1 region

(F: TGCTAGCCGCAGGCATTACT R: CGGGATCAAAGAAAGTTGTGTTT).

Nuclear RNAse-P

(F:GCCTACACTGGAGTCCGTGCTACT, R:CTGACCACACACGAGCTGGTAGAA) gene was used as the nuclear marker gene to standardize mtDNA copy number to diploid chromosomal DNA content according to [2].

Statistical comparison between Gulo and DKO

Most of the comparisons discussed in the paper are between DKO and Gulo KO mice fed with the same dose of ascorbic acid as these Gulo mice are the most appropriate reference. It could be possible to compare DKO with SEPN1KO mice too but, in the majority of cases, the latter do not show any alteration.

Figure Legends

Sup. Figure 1 related to Figure 2

A. ERO1α immunoblot and Ponceau staining of proteins extracted from the gastrocnemii of wild-type and SEPN1KO mice transduced with *AAV2.1-ERO1α* from which the RNAs were extracted for microarray hybridization. Below, the genotype of SEPN1 locus of the gastrocnemii from which the RNAs were extracted for microarray hybridization.

B. Heat map of the genes belonging to the TGF-β pathway that are deregulated in the gastrocnemius of KO/KOERO1.

Sup. Figure 2 related to Figure 3

A. Immunoblot of type I procollagen and ERO1*α* in lysates of WT and KD transiently transfected with *ERO1α* and a hyperactive mutant of *ERO1α (C131A)*. The anti-β-Actin blot serves as loading control.

B. The upper traces show a peak signal for the standard ascorbic acid that is not detected

for the standard DHA indicating that the method is specific for the detection of ascorbic acid and does not detect DHA. The lower traces show a peak signal of Ascorbic acid in cells loaded with DHA, indicating a conversion of the latter to the reduced form of ascorbic acid inside the cells.

Sup. Figure 3 related to Figure 5

A. The upper traces show a peak signal for the standard ascorbic acid that is not detected in the mobile phase indicating that the method is specific for the detection of ascorbic acid. The lower traces show a peak signal of Ascorbic acid in the gastrocnemius of the indicated genotypes, that is absent after treatment with the oxidant H_2O_2 and is greatly reduced after one week of ascorbic acid starvation in Gulo KO mice.

B. Abundance of HSP47 mRNA relative to wt (fold change) measured by quantitative real time PCR in cDNA from gastrocnemii (n=3/5 per each group, bar graphs indicate means ±SEM, differences were examined using a 2-tailed unpaired Student's *t* tests and P values are indicated on the graph).

C. Representative histology of collagen I staining. The insets show the accumulation of collagen in DKO maintained at the low dose of ascorbic acid (scale bar: 100 mm).

Sup. Figure 4 related to Figure 6

A. Western blotting analysis of the rate of new protein synthesis in the gastrocnemii of mice with the indicated genotype and treatment. Puromycin incorporation was detected with anti-puromycin (anti-Pur) antibody, and GAPDH was used as loading control. Graph shows the signal of puromycin of gastrocnemii of four different mice.

B. Real-time PCR quantification of the amount of mtDNA relative to that of RNAse-P, a nuclear gene used as a standard (n=7 per each group, bar graphs indicate means ±SEM,

differences were examined using a 2-tailed unpaired Student's *t* tests and P value is indicated on the graph).

References:

1 Marino, M. *et al.*, SEPN1, an endoplasmic reticulum-localized selenoprotein linked to skeletal muscle pathology, counteracts hyper-oxidation by means of redox-regulating SERCA2 pump activity. *Hum Mol Genet* (24), 1843-1855 (2015).

2 Spinazzola, A. *et al.*, MPV17 encodes an inner mitochondrial membrane protein and is mutated in infantile hepatic mitochondrial DNA depletion. *Nat Genet* 38 (5), 570-575 (2006).

Sup. Fig.1 related to Fig.2

A

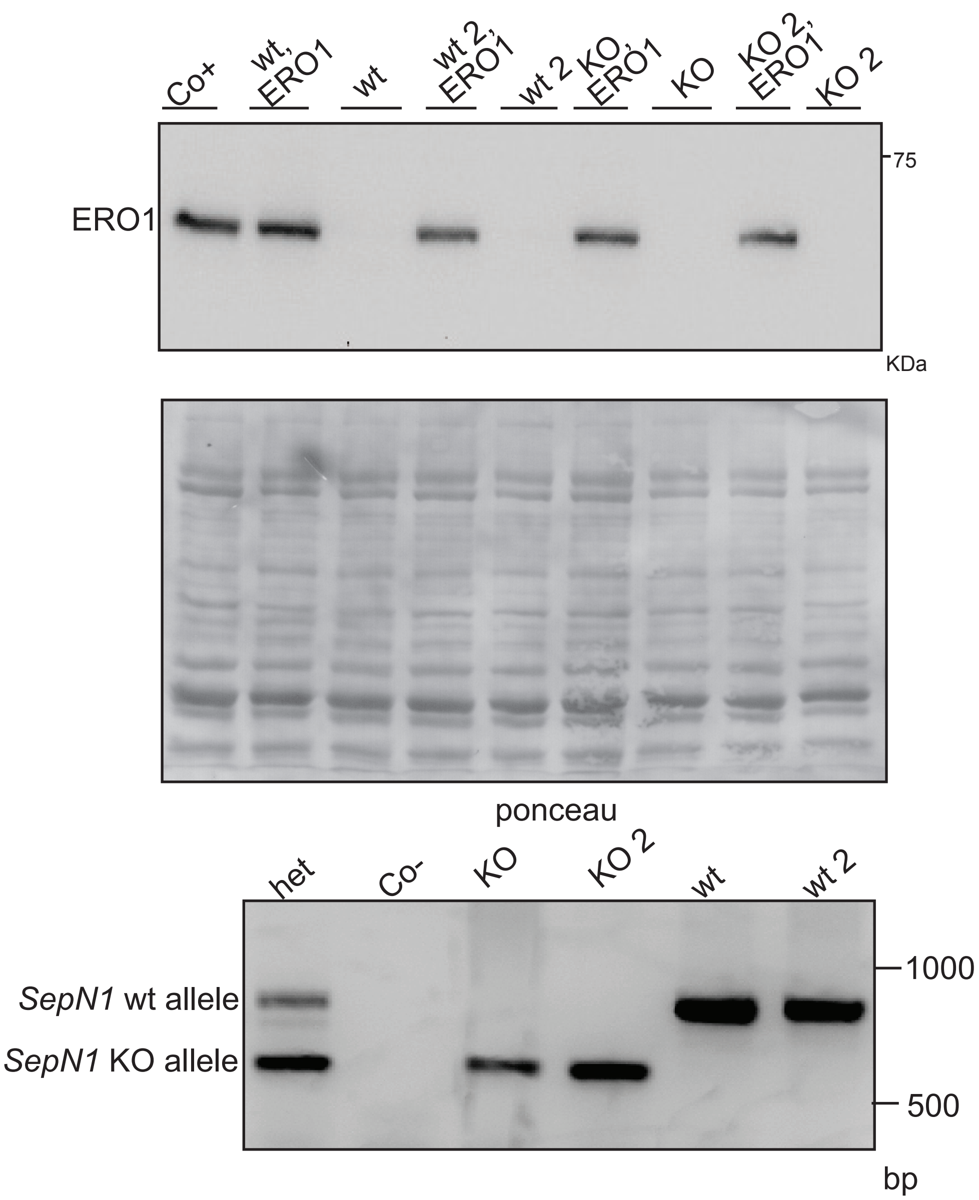

B

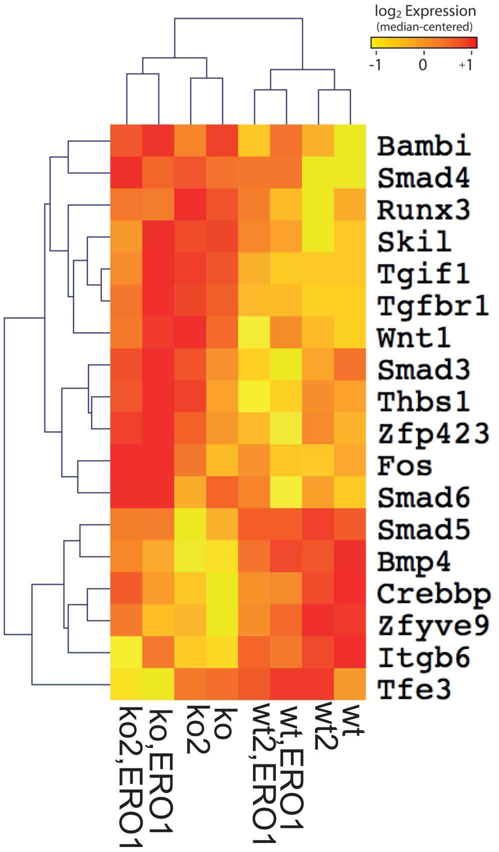

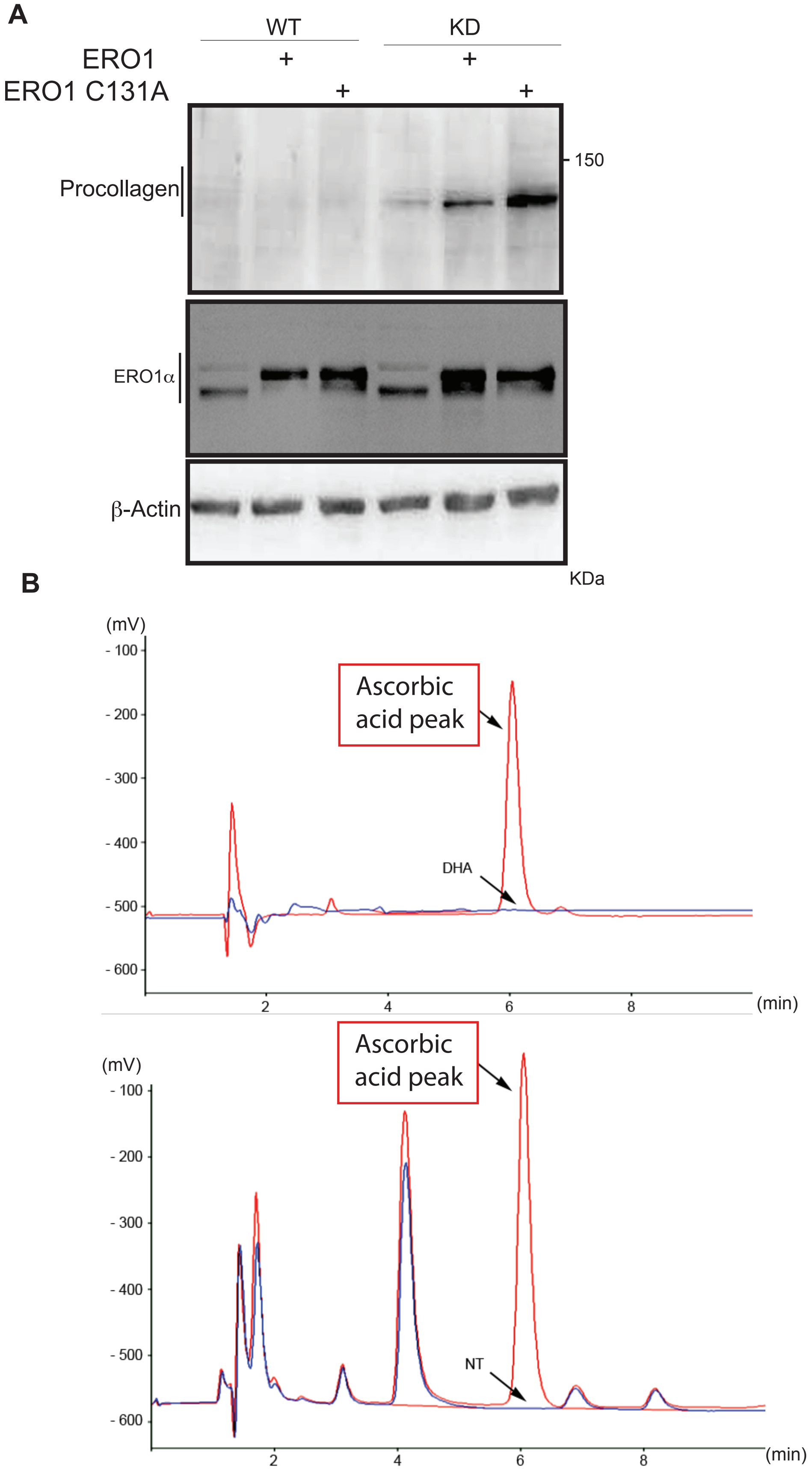

Sup. Fig.2 related to Fig.3

Sup. Fig.3
related to
Fig.5

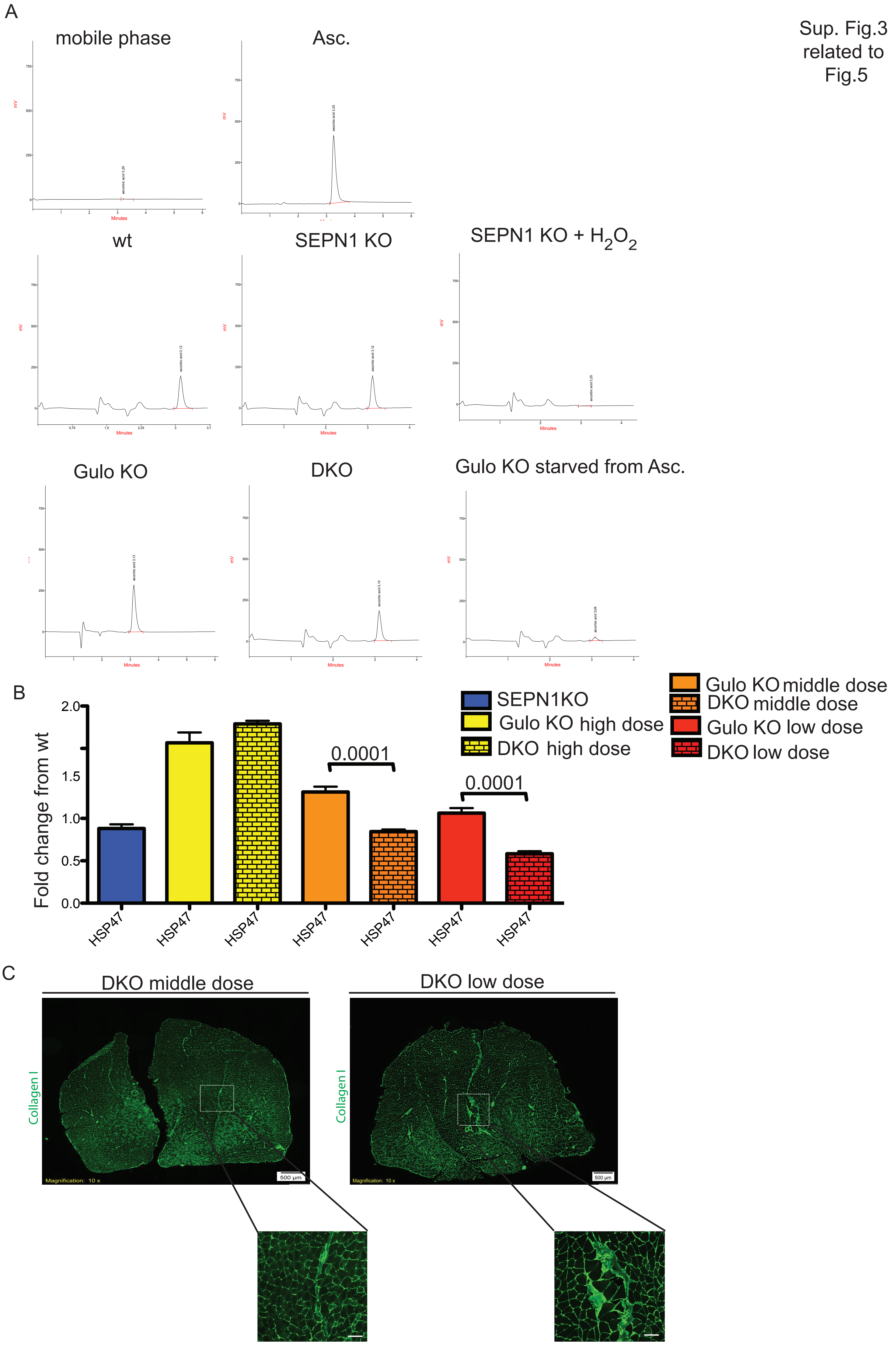

Sup. Fig.4
related to
Fig.6

A

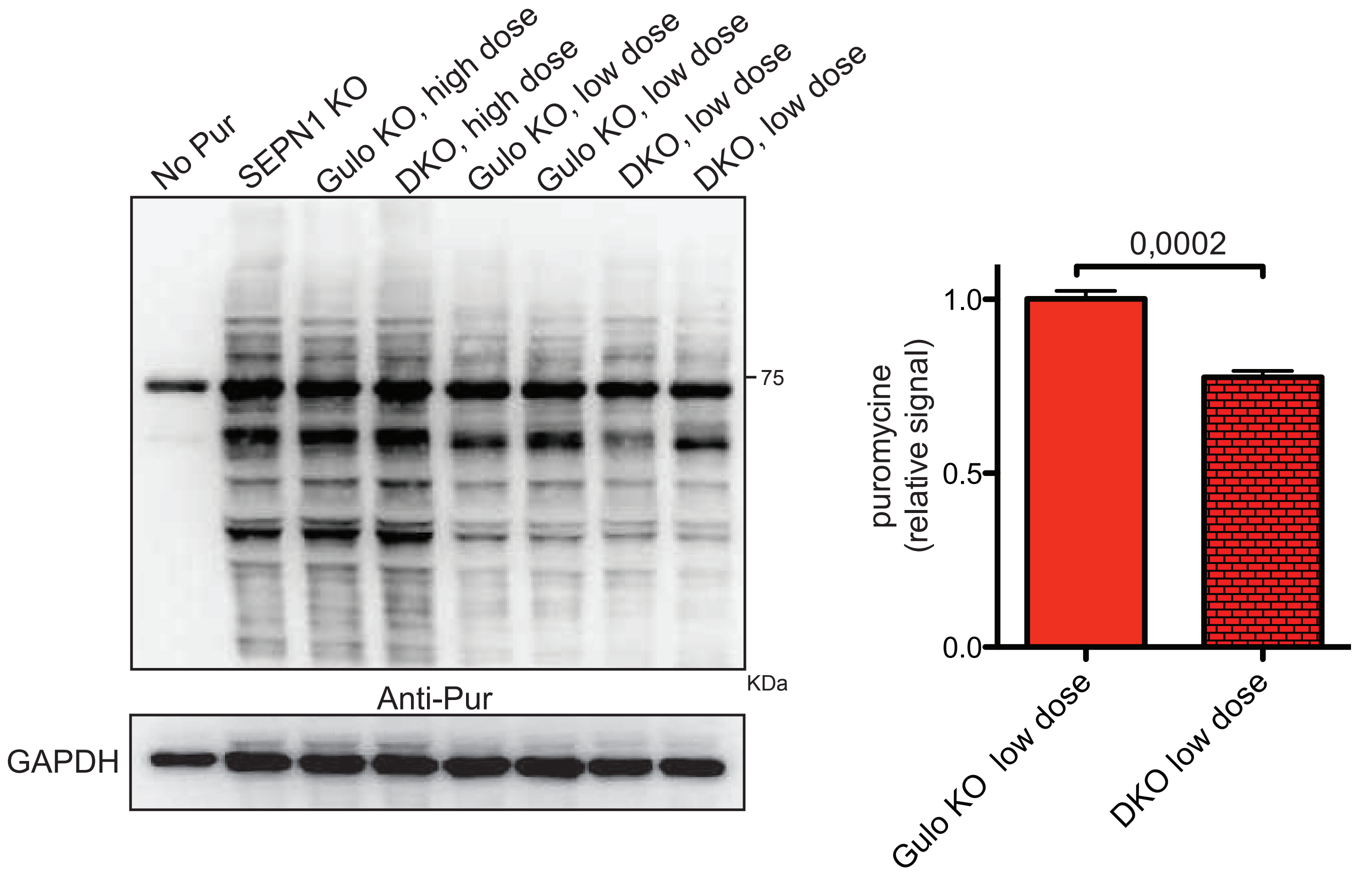

B

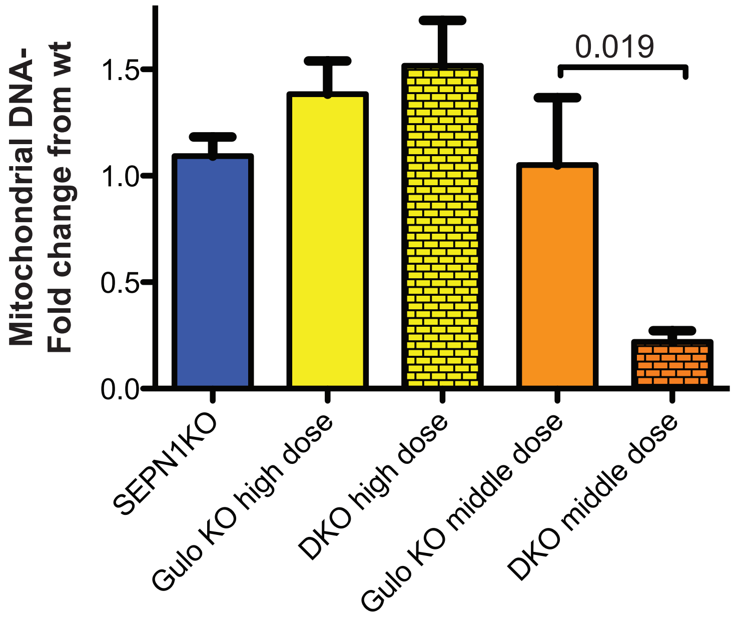

Supplement: Supplementary Information [file srep40993-s1.pdf]
